# Influence of Messa di Voce speed on vocal stability of untrained, healthy subjects

**DOI:** 10.1371/journal.pone.0314457

**Published:** 2025-01-30

**Authors:** Jonas Kirsch, Marie Köberlein, Michael Döllinger, Matthias Echternach

**Affiliations:** 1 Division of Phoniatrics and Pediatric Audiology, Department of Otorhinolaryngology, Munich University Hospital (LMU), Munich, Germany; 2 Division of Phoniatrics and Pediatric Audiology at the Department of Otorhinolaryngology Head & Neck Surgery, University Hospital Erlangen, Friedrich-Alexander-University Erlangen-Nürnberg, Erlangen, Germany; Manipal Academy of Higher Education, INDIA

## Abstract

**Introduction:**

Despite its importance in voice training, comprehensive research into sustained vowel phonation with constant pitch and increasing and decreasing loudness, the so-called Messa di Voce, is lacking. The study examines the laryngeal behavior during Messa di Voce, regarding the impact of the speed of execution on voice stability parameters.

**Materials and methods:**

Nine untrained, healthy subjects (5 female, 4 male) were asked to perform Messa di Voce exercises on the vowel [i:], involving a gradual increase and decrease of volume. During the first task, each phase should take 3 s, whereas in the second task, each phase should take 1 s. Female subjects sang pitch B3 (fundamental frequency *f*_o_ ≈ 247 Hz), and male subjects pitch B2 (*f*_o_ ≈ 124 Hz). Throughout phonation, synchronous recordings were captured through high-speed videolaryngoscopy (HSV), electroglottography, and audio signals. Subsequently, the Glottal Area Waveform was extracted from the HSV data. The tasks’ duration and calculated parameters (including, e.g., Open Quotient (OQ), Closing Quotient (ClQ), Relative Average Perturbation (RAP)), excluding parts of the signal with stationary sound pressure level (SPL), were analyzed with correlation analysis and statistical analysis (Analysis of Variance and subsequent multiple comparisons).

**Results:**

Subjects shortened the requested task length by factor ≈ 0.5. The *f*_o_ remained almost stable for most subjects and tasks. There were strong negative correlations between SPL and both OQ and ClQ. The median RAP appears to decrease towards the SPL apex and then increase again. Statistical effects were shown especially for females during the fast task, which may be due to raised SPL.

**Conclusion:**

There was no specific effect on stability found corresponding to the task’s speed. Also, no major vocal instabilities at a specific sound pressure level were apparent, indicating no transitions as they exist for *f*_o_ regions with registration events.

## Introduction

In the realm of vocal pedagogy and scientific inquiry, Messa di Voce (MdV) stands as a focal point demanding meticulous exploration [1,2]. Messa di Voce, an essential and challenging singing exercise, involves sustaining a pitch while gradually increasing and then decreasing its intensity. Despite its widespread integration into voice training methodologies, the biomechanical underpinnings of MdV have yet to be fully understood.

Messa di Voce exercises can improve voice control, which can help maintaining vocal health by preventing vocal strain [3]. In addition to its practical applications in singing, understanding how subglottic pressure and vocal mechanics work can benefit anyone who uses their voice, e.g., public speakers, actors, teachers and call center staff. Understanding mechanisms of MdV in untrained subjects could contribute to voice therapy and voice training.

The main challenge in executing MdV lies in managing subglottic pressure (*p*_sub_), which influences both main voice characteristics, the intensity and the fundamental frequency (*f*_o_) [4,5]. Consequently, the singer must additionally coordinate other parameters to increase the intensity, such as the adjustment of resonances [6,7] and Maximum Flow Declination Rate (MFDR) [8–10], while likely adjusting the vocal fold configuration to counteract a rise in *f*_o_. An additional difficulty of the task is to provide the required *p*_sub_ while the lung volume decreases constantly [10–13]. Several studies have explored aspects of MdV. It has been observed that with improving MdV control, not only the maximum sound pressure level (SPL) increased, but also voice vibrato was more present, suggesting a possible connection [14,15]. The desired symmetry of the SPL course was rarely found, reporting convex or concave shaped courses and different lengths of the phases, indicating that perception and quantitative measures might differ [16–18]. Regarding the vocal tract, correlations between the SPL course and several articulators were documented, probably influencing the spectral intensity, i.e., lip opening, jaw opening, pharynx width, uvula elevation, and vertical larynx position [3]. Another aspect of MdV is the speed of the execution. For a faster task, the breath dosage for *p*_sub_ is presumed to be less difficult, but vibrato would have less time to evolve and therefore be less present.

In contrast to the *ƒ*_o_ domain which can show instabilities at register transitions (“passaggio”) [6,19–22], it is not known if there can occur similar effects for sound pressure level (SPL). The presented study seeks to explore the impact of the speed of MdV execution on voice stability parameters. Since it is expected that professionally trained singers are able to compensate for difficulties more easily, this study investigates untrained subjects to make potential speed effects visible as a baseline.

## Materials and methods

After receiving approval from the local ethical committee (Medical Ethics Committee of the University of Munich, 20-282), 10 subjects (5 male, 5 female, details in Table 1) were recruited from the clinic’s staff. All subjects were informed in oral (by a qualified medical employee) and written form about the procedure, potential health risks and data handling/protection of the study and that subjects could abort participation at any time. All data was fully anonymized by reduction of the subjects’ identity down to an alphanumeric abbreviation which is only associated with a subject ID and study number. Consent was collected in written form for each: the study procedure itself and data protection/handling.

**Table 1:** Subject number, age and sex of each subject.

Subject number	1	2	3	4	5	6	7	8	9	10*
Age (y)	48.42	26.33	25.17	30.58	29.42	33.92	25.00	28.75	45.75	49.83
Sex	♂	♂	♂	♀	♀	♀	♀	♀	♂	♂

*Excluded later due to one missing crescendo phase.

10 vocally healthy untrained subjects were asked to perform two MdV tasks on the vowel [i:], gradually modulating volume from *piano* (soft phonation) towards *forte* (loud phonation) and back to piano. In the first task (further denoted *slow*), each phase (i.e., *rising* phase from piano to forte and *falling* phase from forte to piano) should last 3 s, and in the second task (further denoted *fast*), each phase should take 1 s. Female subjects were asked to phonate at pitch B3 (fundamental frequency (*f*_o_) ≈ 247 Hz), while male subjects were asked to phonate at pitch B2 (*f*_o_ ≈ 124 Hz). Each subject was provided with the correct pitch played on a piano several times immediately before each task.

Similarly to previous studies [23–25], during phonation, transnasal endoscopic highspeed videos (HSV) were recorded with a Photron Fastcam SA-X2 [26] with an ENF GP Fiberscope [27] and a Storz light source [28] at a rate of 20 kHz and an image resolution of 386 × 320 pixels (monochromatic). In addition, electroglottographic (Glottal Enterprises EG2-PCX2 [29]) and an audio signal (DPA 4061 [30], 4 cm distance) were captured using a NI USB-6251 BNC [31] at a rate of 20 kHz. The audio signal’s amplitude was calibrated using a sound level meter (Tecpel DSL 331 [32]) and the MATLAB software [33]. The HSV sequences were post-processed by means of Fast-Fourier-Transformation, rotation and cropping, as described before [34]. The calculation of the glottal area waveform (GAW) and Phonovibrogram (PVG) [35] from the HSV images was performed with the Glottis Analysis Tools (GAT) software [36] (Version 2020).

Each signal was separated into 100 ms long, non-overlapping windows. The following averaged parameters were calculated using the software Multi Signal Analyzer (MSA) [37] for each window: from the Glottal Area Waveform derived open quotient (OQ_GAW_), electroglottographical open quotient (OQ_EGG_), Closing Quotient (ClQ_GAW_), SPL, Relative Average Perturbation (RAP, version 1 [38]), and EGG derived absolute Sample Entropy [39] (see Table 2 for further details).

**Table 2:** Analyzed parameters and their source. Parameters were computed based on the formulas provided by Schlegel [42].

GAW	EGG	Audio
Closing Quotient (ClQ_GAW_)		
Open Quotient (OQ_GAW)_	Open Quotient (Howard criterion [40], OQ_EGG_)	
	Absolute Sample Entropy	
Relative Average Perturbation (RAP)	RAP	RAP
		Sound Pressure Level (SPL)
Fundamental Frequency (*f*_o_)	*f*_o_	*f*_o_

For the detection of OQ_GAW_ a tolerance threshold of 5% was set. The electroglottographic open quotient (OQ_EGG_) was calculated according to the Howard criterion [40]. For frequency perturbation the RAP for all three voice signals (GAW, EGG, and audio) was measured. Since untrained subjects may show *f*_o_ changes with changes of SPL, an influence on perturbation measurements may be possible. For that reason, RAP has been chosen instead of jitter, because it has been shown to be robust to such as *f*_o_ changes [41].

For correlation analysis, Spearman rank correlation test was used with two-sided hypothesis testing (testing the alternative hypothesis that the correlation is not 0) with a significance level of α=0.05.

This study aimed to analyze voice parameters during the dynamic parts of the recording. To exclude potential stationary SPL phases, only parts between 10% and 90% maximum-to-minimum SPL range were taken into account. To enable direct parameter comparison during SPL rise and fall phase, time courses were normalized in time. Time 0 describes the start of the task, time 1 the point in time in which the SPL reaches its maximum, and time 2 the end of the task. Since rising and falling regions were chosen according to a 10% and 90% minimum-to-maximum threshold, each region has only temporal length of 0.8 instead of 1, to make this visually clearer (although 10% of amplitude rise/fall does not necessarily mean that 10% of the time has passed). Minimum-to-maximum range was calculated individually for rising and falling phases. An example can be seen in S1 Fig.

To directly compare parameters using scatterplots, each subject’s data is approximated to demonstrate correlation. Spearman’s rank correlation is employed, which serves as an indicator of monotonicity. For correlation demonstration, the data is transformed into rank space and a linear fit is performed (see Matlab’s fit() function [43] with linear least squares method and least absolute residual (LAR) method for robustness). However, interpreting a fit in rank space can be challenging. Therefore, the ranked data is projected back into the original data space using the linear fit derived in rank space. This approach allows to visually represent the monotonic relationship in a more interpretable manner. An example can be seen in S2 Fig.

To examine the statistical relationship between measured parameters and slow/fast task or rising and falling SPL phase, an n-way Analysis of Variance (ANOVA, Matlab’s anovan() function [44]) was used. Each parameter value is described by the corresponding subject number, the subject’s sex, the task speed, if the value originates from a rising or falling SPL part and how much time has elapsed since the task has started. To compensate for correlations introduced by measurements taken from the same subject or near each other, the elapsed time and subject number are handled as random variables. The impact of the remaining factors are analyzed individually and in combination with a significance level of α = 0.05 and p-values are adjusted using the Tukey-Kramer procedure [45–47] due to pairwise comparisons and Bonferroni’s method [48,49] to compensate for the number of analyzed parameters per subject (see Matlab’s multcompare() [50]). Only those factors/interactions are analyzed via pairwise comparisons, which are reported as significant by the ANOVA (α = 0.05). All given p-values were already adjusted.

## Results

**Fig 1:**
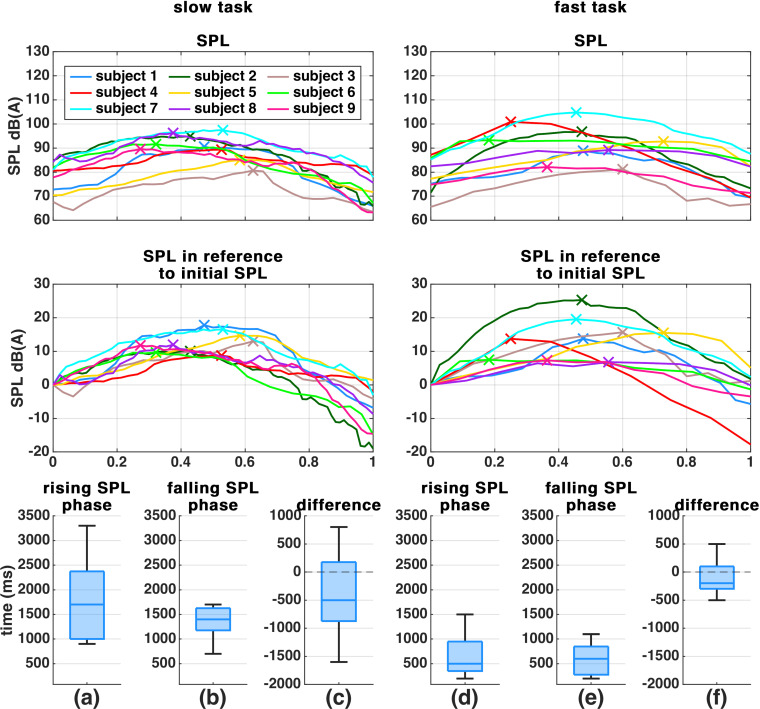
Absolute SPL course and relative SPL in reference to initial SPL (row 1 and 2), together with SPL rise and fall phase durations (row 3). The left column shows the slow MdV task (3 + 3 s) and the right column shows the fast MdV task (1 + 1 s). SPL courses were normalized in time to improve comparability. Phase durations were chosen according to 10% and 90% SPL thresholds. The SPL apex is marked with a cross.

Nine out of ten subjects completed both MdV tasks successfully. Since subject 10 missed a crescendo part during the fast MdV, the (male) subject was excluded from further analysis (S3 Fig shows the SPL course of subject 10). Row 1 and 2 of Fig 1 show the SPL course of all subjects. The SPL apex positions differed between subjects and task and many subjects ended the task with considerably lower SPL compared to the beginning, independent of the speed (slow/fast). In comparison to each other, the subjects’ SPL spread more during the fast task than in the slow task. S1 Table provides age statistics of all remaining subjects.

### SPL phase durations

Fig 1 a, b, d and e show the SPL rise and fall phase durations during both MdV tasks. During the slow task the median rise and fall times were 1.7 s and 1.4 s, whereas during the slow task, the median rise and fall times were 0.5 s and 0.6 s.

Fig 1 c and f show each subjects’ SPL phase duration differences. Rising and falling phases were unsymmetric: during the slow task, the falling phase took in the median about 500 ms longer than the rising phase. Similarly, during the fast task, the falling phase took 200 ms longer than the rising phase. Note the difference to d and e, which seem to suggest that the falling phase was 100 ms shorter than the rising phase during the fast task.

S4 Fig shows the total task durations, which includes all potential stationary SPL parts.

### Fundamental frequency course

**Fig 2:**
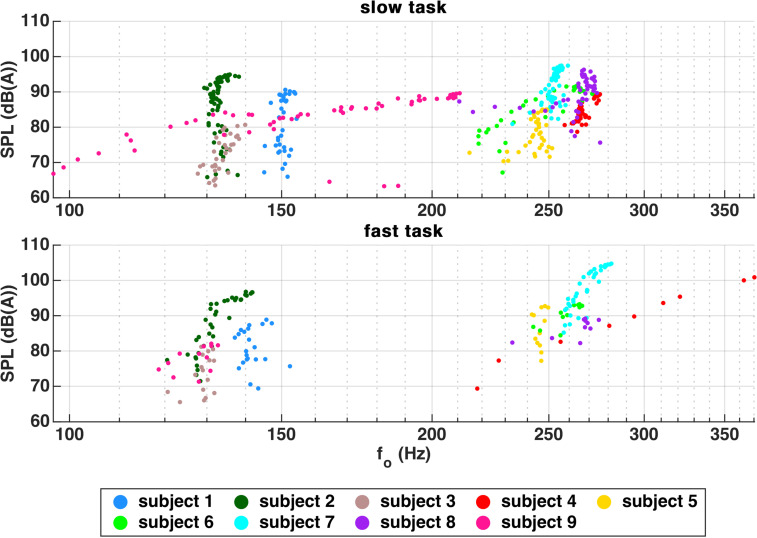
Scatter plot of *f*_o_ (derived from EGG) vs. absolute SPL. Most subjects were able to perform MdV without major *f*_o_ changes. Only subject 4 (during the fast task) and 9 (slow task) in-/decreased SPL simultaneously with *f*_o_.

Fig 2 shows a scatter plot of *f*_o_ (derived from EGG) compared to SPL. Most subjects were able to keep their *f*_o_ stable. Only subject 9 showed major *f*_o_ changes during the slow task and subject 4 during the fast task.

### Closing Quotient (ClQ)

**Fig 3:**
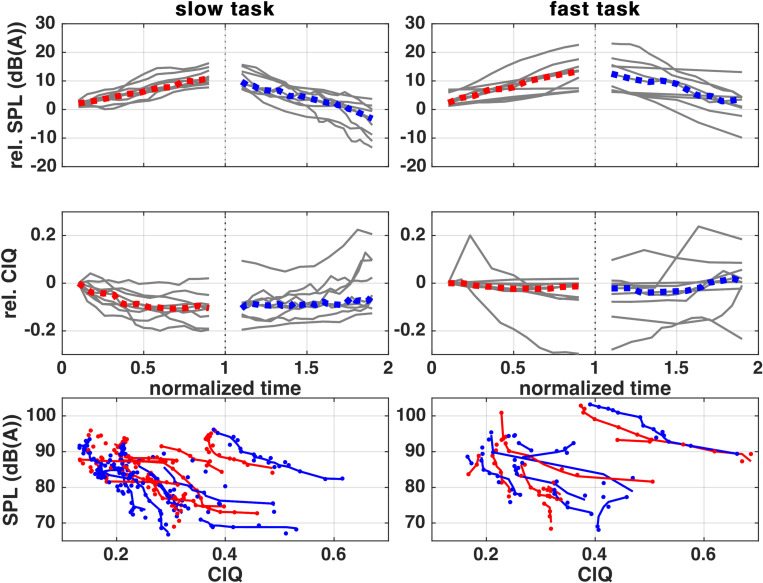
Relative SPL course (upper row) and relative ClQ_GAW_ (middle row) during rising and falling SPL phase. The lower row shows absolute SPL compared to ClQ_GAW_ with backprojected linear fit of ranked source data. The subjects’ individual courses are depicted as grey, whereas the median course is marked with color.

Fig 3 shows relative SPL and relative ClQ_GAW_ during crescendo and decrescendo phase. It can be seen in row 1 that all subjects increased and decreased their SPL and that the speed and linearity of SPL in-/decrease was comparable between subjects with normalized time.

During the slow task, the median SPL change was +9 dB(A) and −13 dB(A). During the fast task, the subjects median SPL change was +11 dB(A) and –9 dB(A).

In both tasks, the median of the relative ClQ_GAW_ develops inverse proportionally to the SPL. The median ClQ_GAW_ change was −0.11/ +0.06 during the slow task and −0.01/ +0.02 in the fast task, respectively. S2 Table presents all significant correlation values, which consistently demonstrate a strong negative correlation during the slow task. During the fast task, only 3 or 2 subjects show a significant correlation during the rising/falling SPL phase. S3 Table contains the linear fit parameters used for the back-projected lines in row 3 of Fig 3.

The pairwise comparisons reveal that the ClQ_GAW_ is significantly bigger for females than for males (0.273 < 0.337), and that it is smaller during the slow task than in the fast (0.272 < 0.338), and furthermore that females have a higher ClQ_GAW_ during the fast task compared to females during the slow or males during both tasks (0.393 > 0.281; 0.263; 0.281). S5 Fig shows the boxplot of the ClQ_GAW_ subdivided by the factors sex, task speed and SPL phase.

### Open Quotient (OQ)

**Fig 4:**
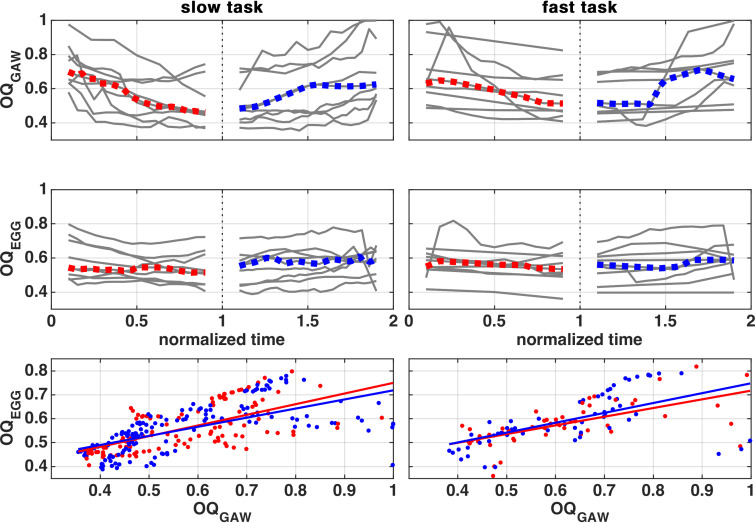
OQ_EGG_ and OQ_GAW_ during rising and falling SPL phase. Row 3 shows a scatter plot and linear fit of both values during SPL rise and fall phase. Fit parameters are shown in S4 Table. The subjects’ individual courses are depicted as grey, whereas the median course is marked with color.

Fig 4 shows the course of OQ_GAW_ and OQ_EGG_ during rising and falling SPL phases, as well as the comparison of both OQs during both tasks, separated into rising and falling SPL phases.

The median OQ_EGG_ course shows minor changes (−0.02/ +0.05) during the slow task and also in the fast task (−0.02/ +0.03). S5 Table contains each subject’s correlation coefficients. Most subjects show a significant and mostly negative correlation between OQ_EGG_ and SPL during the slow task (8 in rising/ 5 in falling SPL phase), but during the fast task, only few subjects show a significant correlation (3 during rising and 1 during falling SPL phase).

The median OQ_GAW_ course shows great changes (−0.24/ +0.15) during the slow task and in the fast task (−0.12/ +0.14). S6 Table shows that most subjects show a significant and highly negative correlation between OQ_GAW_ and SPL during the slow task (6 in rising. 8 in falling SPL phase), and during the fast task, 5 subjects show a significant correlation during the rising and 1 during the falling SPL phase.

By pairwise statistical analysis it was found that OQ_EGG_ and OQ_GAW_ are smaller for males than for females (OQ_EGG_: 0.551 < 0.591, OQ_GAW_: 0.554 < 0.637) and smaller in the slow task than in the fast task (OQ_EGG_: 0.555 < 0.588, OQ_GAW_: 0.567 < 0.624). In addition, the OQ_GAW_ is bigger for females during the fast task, than for females in the slow task and males in both tasks (0.692 > 0.583; 0.552; 0.555). S6 Fig contains a boxplot for both OQs subdivided by factors sex, task speed and SPL phase for better data visualization.

### Phonovibrograms (PVGs)

**Fig 5:**
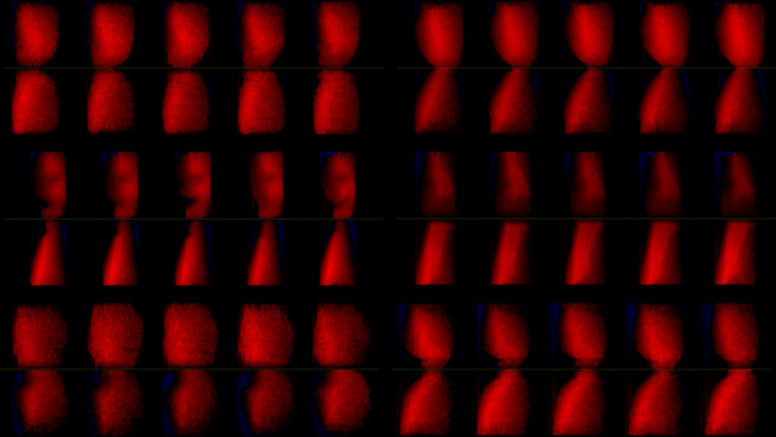
PVGs of subject 1 at task start, near SPL apex and near the end of the task (from top to bottom). The left column originates from the slow task, the right from the fast task. The PVGs were normalized in time to make cycle comparison easier. In consequence, the fundamental frequency cannot be compared. The brighter the red, the bigger the vocal folds’ distance from the glottis midline.

**Fig 6:**
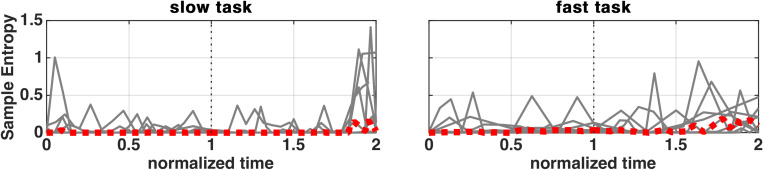
Sample Entropy (derived from EGG) before and after maximum SPL. The subjects’ individual courses are depicted as grey, whereas the median course is marked as red.

In order to check for the existence of rapid changes in vocal fold oscillation patterns, Phonovibrograms (PVGs) were calculated for each subject from the glottal area (Fig 5) and the Sample Entropy was calculated for the whole EGG signal (without the use of SPL thresholds to look for potential peaks of sample entropy during maximum SPL), which is shown exemplified by subject 1 in Fig 6.

### Absolute sample entropy

The EGG derived median Sample Entropy course shows minor changes during the slow task (0.000/ +0.021) and also in the fast task (+0.035/ +0.060). During the fast task, there was an increase of the Sample Entropy towards the SPL apex and in both tasks, there was an increase towards the end of the task.

S7 Table shows that if there was a significant correlation between Sample Entropy and SPL, it would be mostly negative. Statistically, only during the slow task, there was a significant correlation of 2 and 4 subjects (during and rising/falling SPL phase respectively).

### Relative Average Perturbation (RAP)

**Fig 7:**
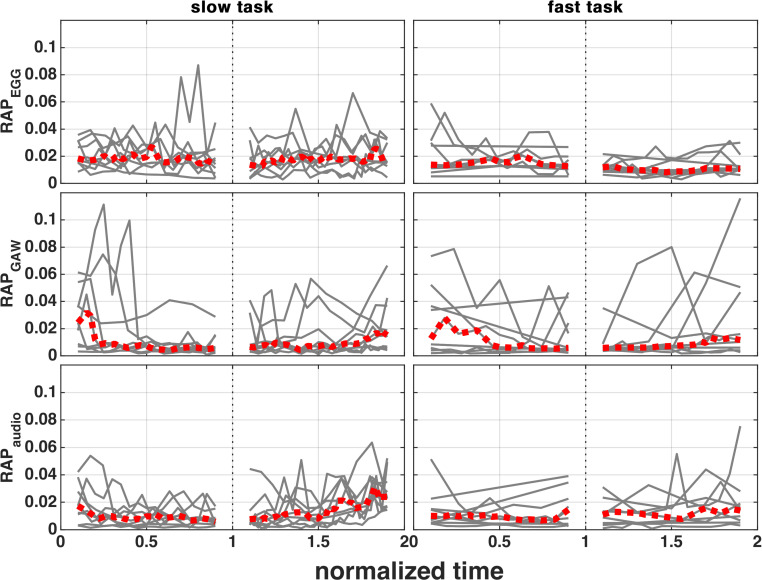
RAP_EGG_, RAP_GAW_ and RAP_audio_ during rising and falling SPL phase. The subjects’ individual courses are depicted as grey, whereas the median course is marked as red.

For further stability analysis, the RAP was calculated for audio, EGG and GAW signals during both tasks (see Fig 7).

The median RAP_audio_ course shows medium changes (−0.011/ +0.016) during the slow task and also in the fast task (+0.005/ +0.003). The median RAP_EGG_ course shows minor changes (−0.004/ +0.002) during the slow and the fast task (−0.001/ −0.001). The median RAP_GAW_ course shows more pronounced changes (−0.018/ +0.008) during the slow and the fast task (−0.007/ +0.006). S8, S9 and S10 Tables contain the correlation values between RAP and SPL measurements for each subject. During the slow task, either 4 or 6 subjects showed significant correlation values in both SPL phases, but only at maximum 2 subjects show significant correlation values. However, there is a consistent (except one) decrease of the median RAP towards the SPL apex and in all but one case a rise of the median RAP towards the end of the task.

Pairwise statistical comparisons showed that for RAP_GAW_, RAP_audio_ and RAP_EGG_ males have a lower RAP than females (0.10 < 0.18; 0.10 < 0.16; 0.16 < 0.20). In addition, the RAP_EGG_ is bigger during the rising SPL phase than in the falling (0.20 > 0.16) and that the slow task has higher values than the fast task (0.19 > 0.16). S7 Fig shows the boxplots for each RAP type and is maximally grouped by all ANOVA factors.

## Discussion

The present study aimed to investigate the impact of speed on the execution of MdV by examining voice parameters based on audio, EGG and GAW. The study found that most subjects were able to increase and decrease their SPL during the MdV exercise, while keeping the *f*_o_ almost stable. This contradicts the expectation that untrained healthy subjects may struggle at keeping their *f*_o_ stable. There were only minor effects of execution speed on the stability during MdV.

The subjects’ rising and falling SPL phases took much shorter than the subjects were instructed. The target duration of each phase was 3 s during the slow task and 1 s during the fast task. The actual rise and fall time taken was about half the requested time. The median total task recording time including potential SPL stationary parts took about 2/3 of the requested time, which indicates, that 1/6 of the requested time was over or above the used 10%/ 90% threshold. This may be due to nervosity or bad sense of time during endoscopy (enforced by the fact that the subjects were not professional singers).

The SPL phases were slightly asymmetrical in length with an in median 500 ms/ 200 ms longer SPL rise than SPL fall phase during the slow/ fast task. This may indicate that subjects realized during the experiment, that they have to perform longer than expected. This may also have introduced the tendency to end with a smaller SPL compared to the start.

The ClQ_GAW_ developed inversely proportional to the SPL in both slow and fast tasks. This finding is consistent with the existing literature [51,52], which suggests that ClQ_GAW_ is negatively correlated with the SPL due to a steeper MFDR. However, it is noteworthy that this effect, which could be caused by a rise of *p*_sub_, had no obvious effect on *f*_o_. It might be that the subjects, although they had no professional training, have generated the SPL increase by resonance adjustments, which is frequently found in professional subjects (see Burk et al. [3]). The statistical analysis showed that females’ and the fast task’s OQ values are in each case statistically higher than for males or the slow task respectively. Since the double grouped data of females during the fast task are also much higher than the other three subgroups, the statistical effect on the main factors may be solely driven by that subgroup. This group seemed to have a specifically raised ClQ_GAW_ and SPL.

The study found significant correlations between OQ_EGG_ and SPL, as well as OQ_GAW_ and SPL during the fast task, which were mostly negative. However, only a few subjects showed a significant correlation during the slow task. The correlation between OQ and SPL is consistent with existing literature [53,54], and may be caused by an increase of subglottic pressure [55–57], which may prompt the glottis to close with greater force to enhance glottal resistance against the subglottic pressure. As a side observation, it can be seen that the agreement of OQ_GAW_ and OQ_EGG_ is good when OQ_GAW_ *<0.08*, which was also found similarly by Echternach et al. [58]. The statistical analysis showed the same pattern as for the ClQ_GAW_ and the effect may have the same origin as for the ClQ_GAW_.

The RAP_audio_, RAP_EGG_ and RAP_GAW_ also showed minor changes during both slow and fast tasks. Most subjects showed a significant and mostly negative correlation between RAP and SPL during the slow task. During the fast task, there was only one subject in each SPL phase with a significant correlation to RAP_GAW_. In summary, this suggests that the speed of performing MdV might have a small impact on the voice perturbation. The statistical analysis showed in general higher values for females compared to males for all RAP types. It should be mentioned here that *f*_o_ for male voices was an octave lower than female voices, thus the double number of pictures/ samples were available for a glottal cycle, which could in part explain the lower values for male voices. However, since values were averaged for 100 ms windows, the compared number of mean values is the same, but those mean values may be more stable for the male subjects. The RAP_EGG_ was significantly higher during the rising than for falling SPL phase, which may be due to instability caused by the force which is needed for SPL increase. Surprisingly, the RAP_EGG_ values seemed to be higher for the slow task compared to the fast task. The reason could also be that this was observed by chance, since the significance level was 0.05 and there were in summary not many significant p-values and this type only for RAP_EGG_.

The number of significant correlations observed in the study varied depending on the parameter and the speed of the task. For instance, during the slow task, most subjects showed a significant and mostly negative correlation between the OQ_EGG_ and the SPL. However, during the fast task, only a few subjects showed a significant correlation. This suggests that the speed of performing MdV might have a more pronounced effect on the vocal fold opening and closing patterns in some individuals than others.

However, it is also crucial to consider the potential for measurement error or variability in individual responses when interpreting these results.

## Limitations

The duration boxplots in Fig 1 show that subjects may have misinterpreted the task to perform 1 s or 3 s rising/falling SPL phases during the MdV. Instead, each phase took about half the duration which was asked for (i.e., e.g. 0.5 s instead of 1 s rising SPL during the slow task). Nevertheless, the fast task still remained shorter than the slow task. Another explanation for the short SPL rising/falling phase may be that subjects needed some time to start rising SPL at the beginning/ start falling after the SPL peak, and/or performed the rise/fall faster than the requested duration and/or lingered near the high SPL peak (resulting into an SPL plateau in that case).

Another limitation are the room acoustics. Since measurements were acquired in a rather small treatment room, minor reflections, room modes or even resonating sounds caused by loud phonation could have influenced the subjects. Also, the SPL was used to identify the apex point in time, but the perceived loudness might differ from this. In addition, since two subjects did not keep the *f*_o_ stable, this effect may be enforced.

The specified task durations were chosen arbitrarily, which means a comparison between both task types may not contain the effects which might occur at other speed instructions. Further, due to the extensive experimental setup, the number of subjects was rather small, statistical results have only limited resilience. Also, transnasal endoscopic measurements can impact the performance of the subjects. On the other hand, subjects may even get used to endoscopy, as reported by Dejonckere et al. [59].

Subjects who altered (increased) their pitch may face additional difficulties to execute the task since an increase in pitch is often accompanied by a narrowing of the vocal tract in untrained subjects (e.g., larynx position rises with increasing *f*_o_ [60–62]).

Normally, a MdV is performed from piano to forte and back, but this procedure showed that the SPL curves get uneven concerning the range in rising and falling phase (often softer at the end than at the beginning). It may be tried out to reverse the order of SPL in- and decrease or to instruct to perform two MdV in sequence without stopping phonation. This would give the possibility to be more independent of individual difficulties concerning the sequence of SPL rise and fall or give the opportunity to analyze those difficulties. Alternatively, direct visual feedback could be given to help improving comparability of SPL range and shape during rise and fall phase.

Parameters were compared using a normalization in time according to the recordings’ SPL apex. This does not account for different types of SPL in-/decrease shapes, like shown by, e.g., Collyer et al. [16], which may distort comparison.

ANOVA was performed using the main factors corresponding to the subjects’ sex, the SPL phase and the task speed. The model could be tested against models with additional factors like *f*_o_, SPL and another nearly arbitrary number of subjects, which may increase the model precision in some way, but may also lead to overfitting of the underlying data. At the same time, all statistics were performed on a relatively low number of samples.

## Conclusion

This study investigated the impact of execution speed on voice parameters during MdV in untrained subjects. The correlation of SPL to OQ, ClQ_GAW_ and RAP was, if significant, mostly negative. Nevertheless, there were no SPL breakpoints found as it is known for vocal register transitions. There were no great effects on voice stability found that are specifically related to the execution speed of MdV.

## Declaration of Generative AI and AI-assisted technologies in the writing process

During the preparation of this work the author(s) used Microsoft Copilot to check language, since the authors were non-native speakers. After using this tool/service, the author(s) reviewed and edited the content as needed and take(s) full responsibility for the content of the publication.

## Supporting information

S1 Fig SPL thresholding example. The 10% and 90% values are calculated for rising and falling phase separately. The borders mark the first value greater 10% and smaller 90%. Rising phase range is 100 dB, resulting in thresholding values of 10 dB and 90 dB. Falling phase has range 120 dB, resulting into borders at 88 dB and −8 dB.
(TIF)

S2 Fig Example of noisy monotonous data, which is transformed into rank space, linearly fit and backprojected into data space. (TIF)

S3 Fig SPL course of subject 10, which was excluded from analysis since it misses a crescendo part during the fast MdV task. (TIF)

S4 Fig Total task duration boxplots. Horizontal black dashed lines mark the target task duration (3 + 3 = 6) s and (1 + 1 = 2) s. Task median durations were 3900 s and 1200 s.
(TIF)

S5 Fig ClQ_GAW_ boxplot visualization for statistical pairwise comparisons of ANOVA factors sex, task speed and SPL phase. (TIF)

S6 Fig OQs boxplot visualization for statistical pairwise comparisons of ANOVA factors sex, task speed and SPL phase. (TIF)

S7 Fig Boxplot visualization of RAP values for statistical pairwise comparisons of ANOVA factors sex, task speed and SPL phase. (TIF)

S1 Table Age data and statistics of all 9 successful subjects. (XLSX)

S2 Table Spearman rank correlations of each subject between SPL and ClQ_GAW_. Significant p-values (<0.05) are marked in green.
(XLSX)

S3 Table Linear fit parameters of SPL and ClQ_GAW_ in rank space. If the line has negative slope, the cells are marked in green.
(XLSX)

S4 Table Linear fit parameters of OQ_EGG_ and OQ_GAW_. (XLSX)

S5 Table Spearman rank correlations of each subject between SPL and OQ_EGG_. Significant p-values (<0.05) are marked in green.
(XLSX)

S6 Table Spearman rank correlations of each subject between SPL and OQ_GAW_. Significant p-values (<0.05) are marked in green.
(XLSX)

S7 Table Spearman rank correlations of each subject between SPL and Sample Entropy. Significant p-values (<0.05) are marked in green. NaN values correspond to correlation measurements in which at least one of both variables show no variation, resulting into a non-valid correlation value.
(XLSX)

S8 Table Spearman rank correlations of each subject between SPL and RAP_audio_. Significant p-values (<0.05) are marked in green.
(XLSX)

S9 Table Spearman rank correlations of each subject between SPL and RAP_EGG_. Significant p-values (<0.05) are marked in green. NaN values correspond to correlation measurements in which at least one of both variables show no variation, resulting into a non-valid correlation value.
(XLSX)

S10 Table Spearman rank correlations of each subject between SPL and RAP_GAW_. Significant p-values (<0.05) are marked in green.
(XLSX)

S1 Text PDF excerpt from the Glottis Analysis Tools [36] containing the explicit formulas used for parameter calculation. Those formulas are also used in the Multi Signal Analyser [37].
(PDF)
