# Supplementary figures and images for: Influence of Messa di Voce speed on vocal stability of untrained, healthy subjects
Source: PLoS One. 2025 Jan 30;20(1):e0314457. doi: 10.1371/journal.pone.0314457 (PMC11781711; doi:10.1371/journal.pone.0314457)

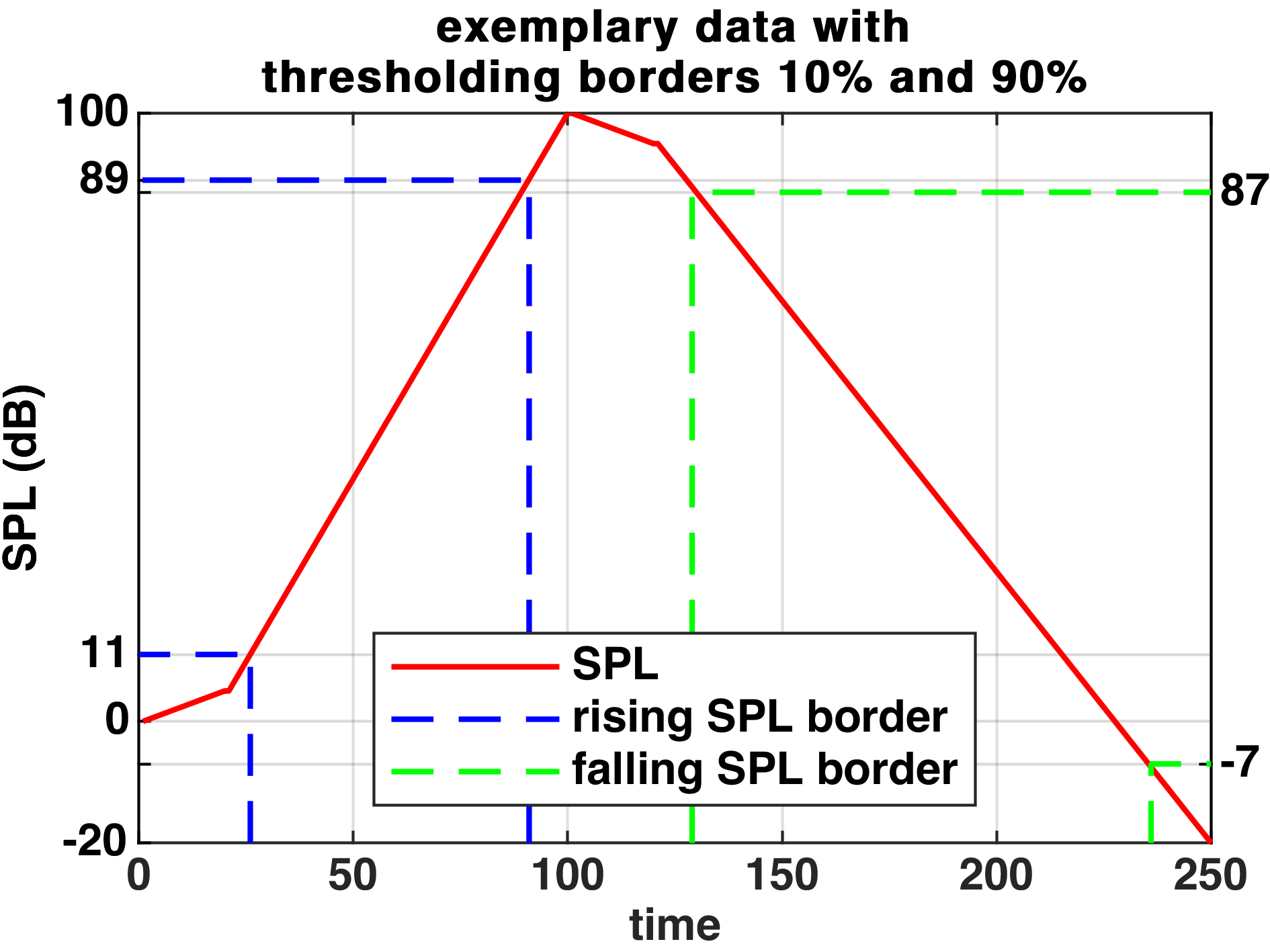

Supplement: S1 Fig — The 10% and 90% values are calculated for rising and falling phase separately. The borders mark the first value greater 10% and smaller 90%. Rising phase range is 100 dB, resulting in thresholding values of 10 dB and 90 dB. Falling phase has range 120 dB, resulting into borders at 88 dB and −8 dB. (TIF) [file pone.0314457.s001.tif]

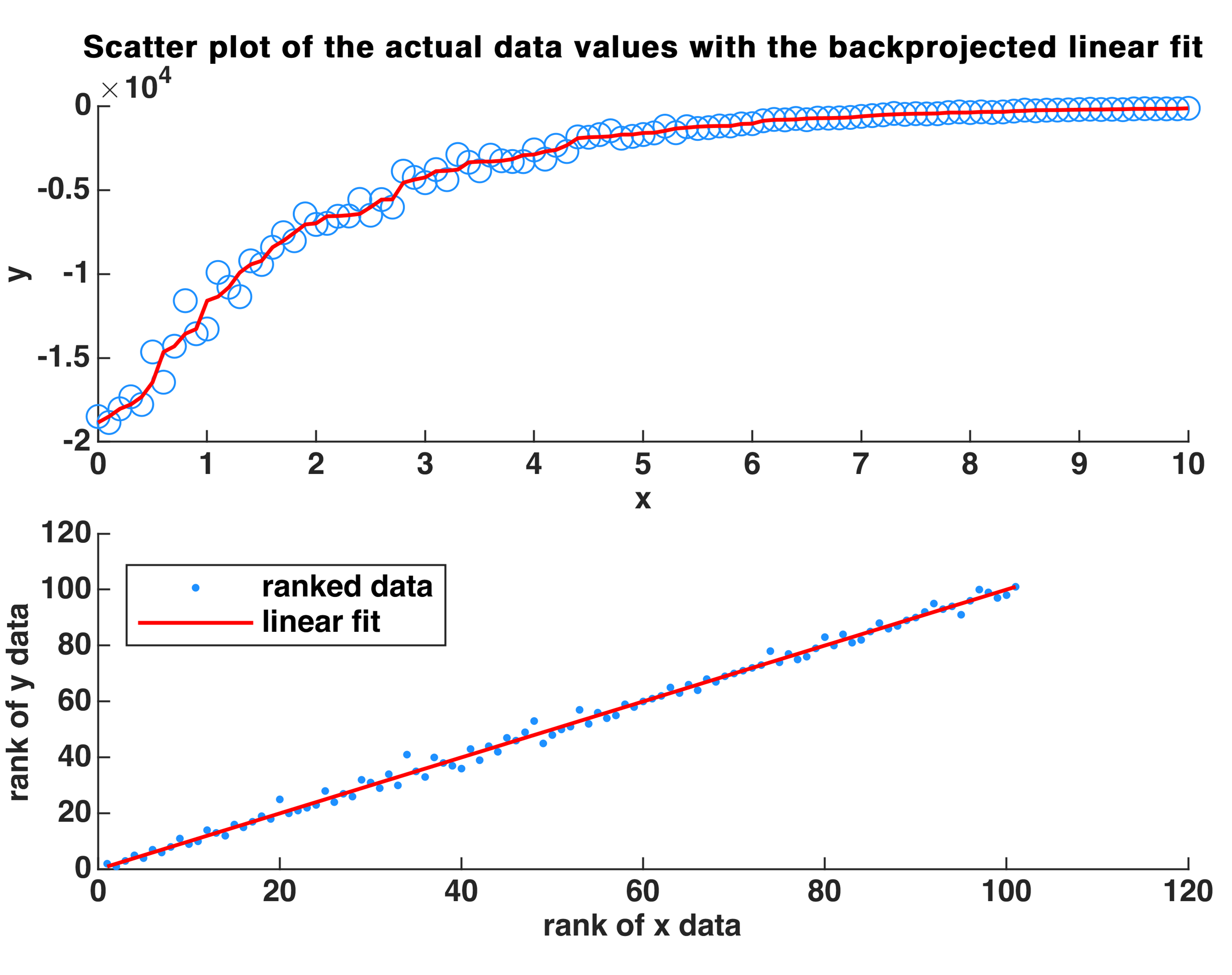

Supplement: S2 Fig — (TIF) [file pone.0314457.s002.tif]

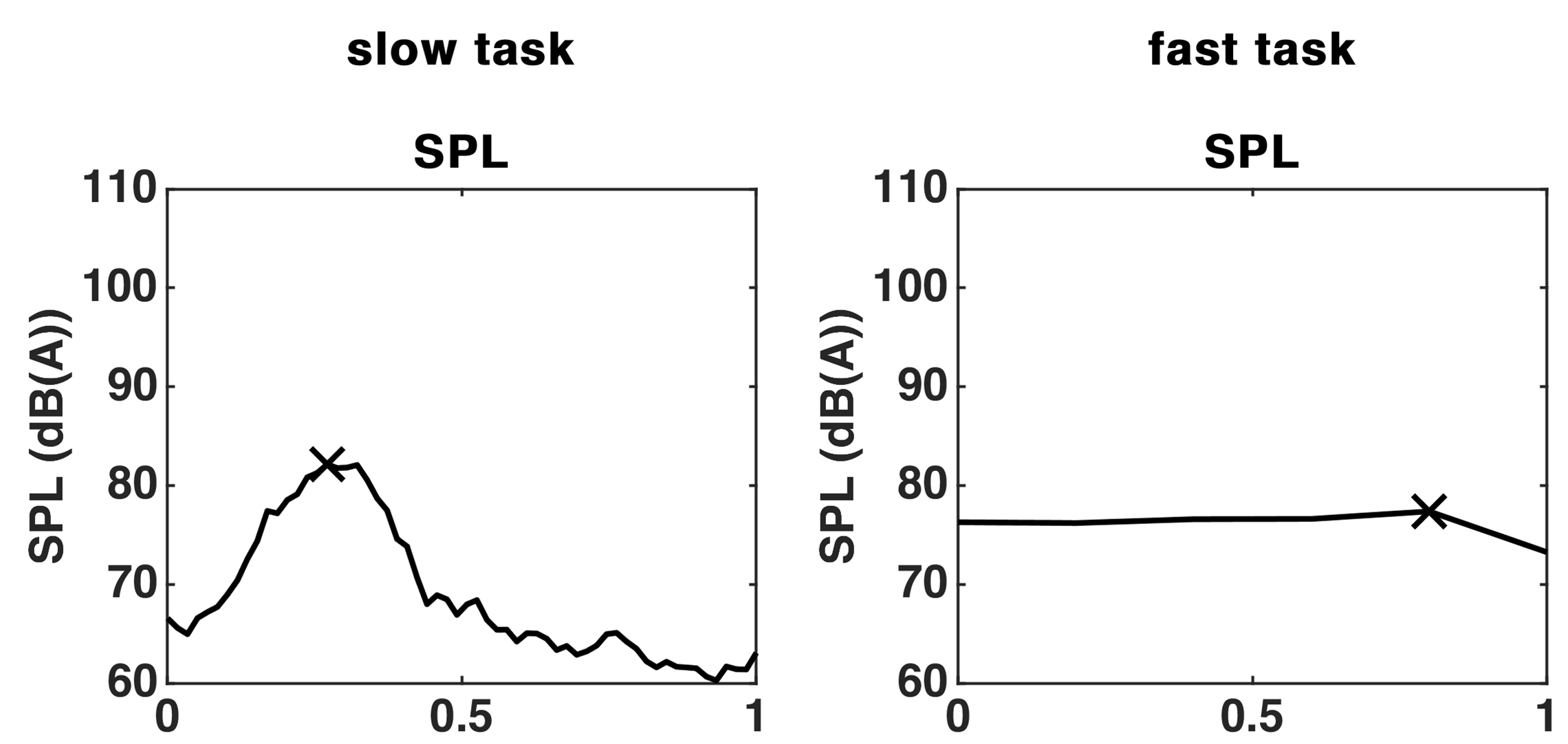

Supplement: S3 Fig — (TIF) [file pone.0314457.s003.tif]

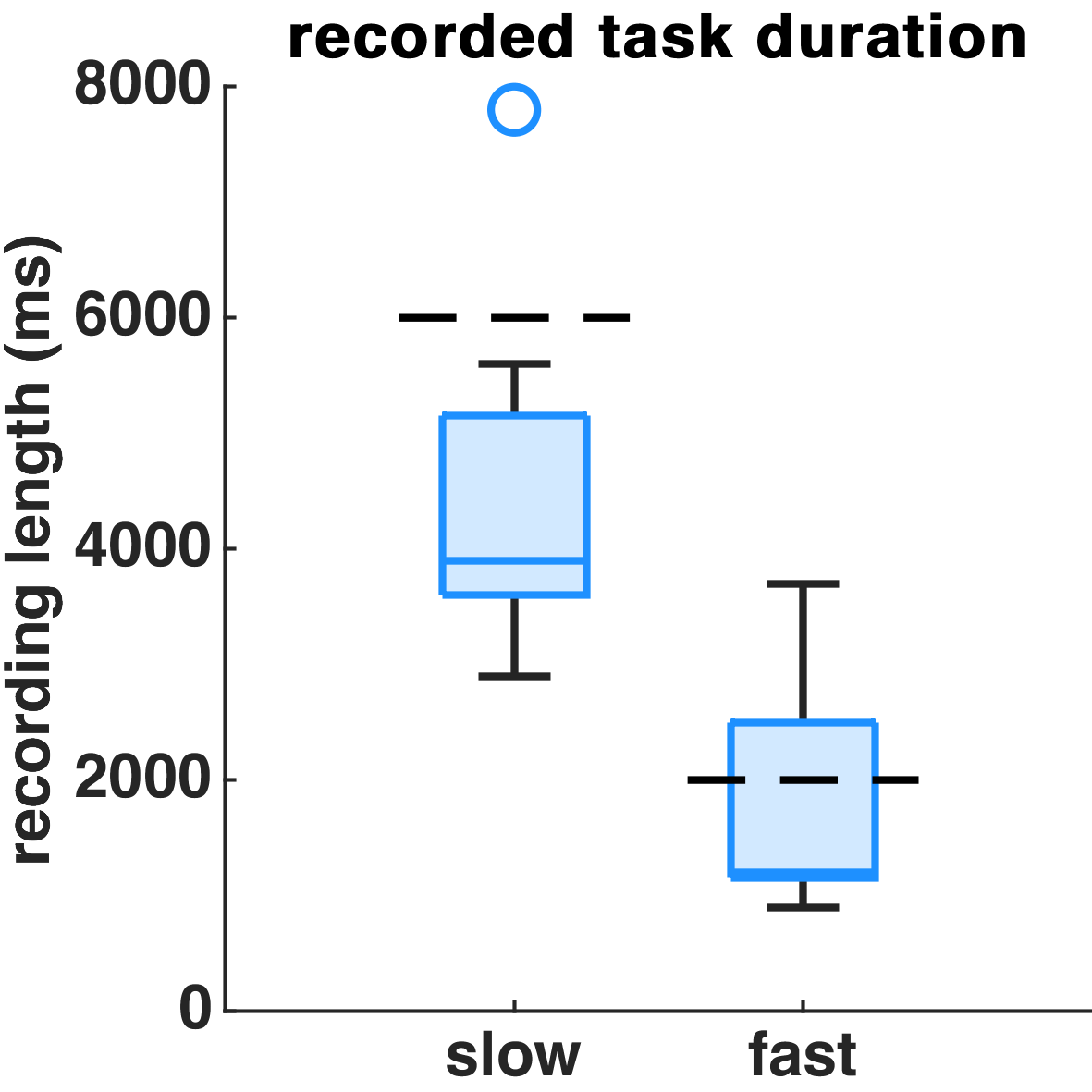

Supplement: S4 Fig — Task median durations were 3900 s and 1200 s. (TIF) [file pone.0314457.s004.tif]

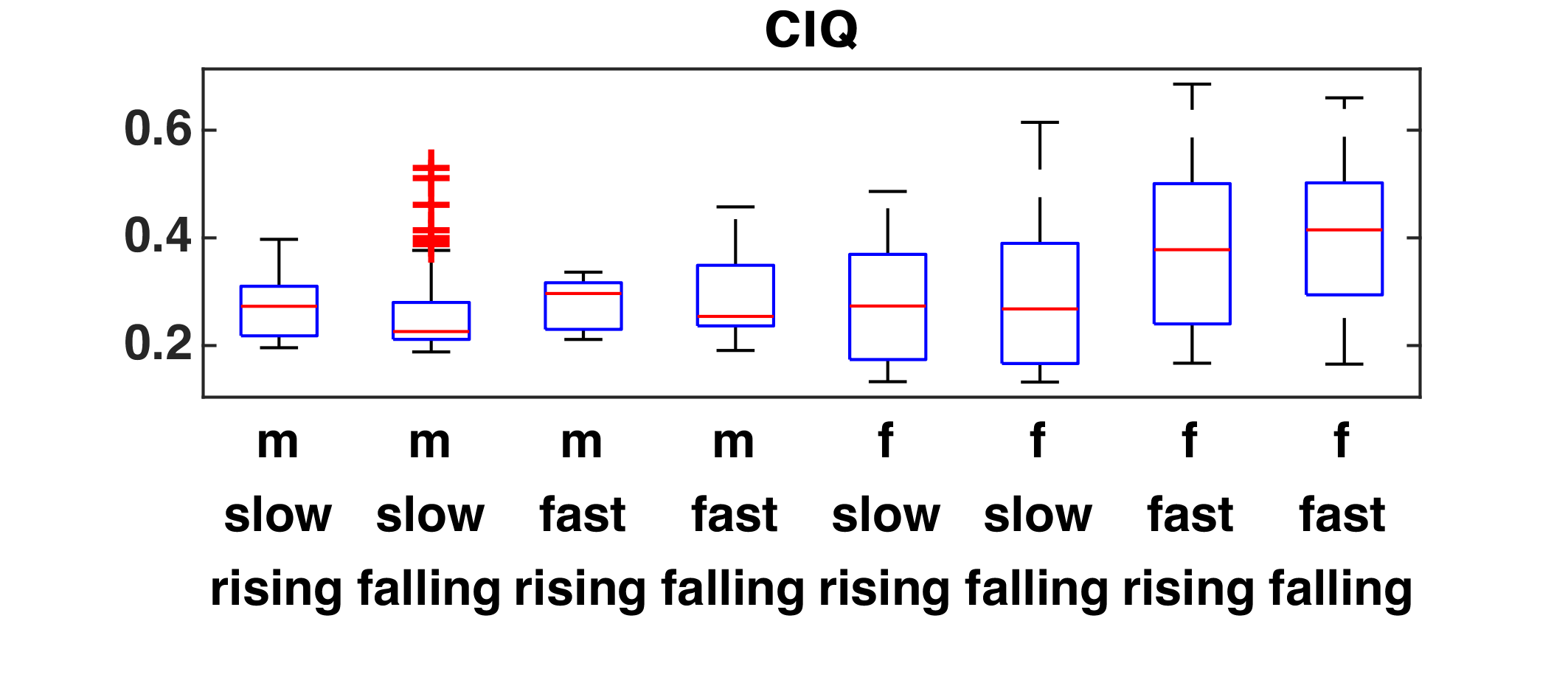

Supplement: S5 Fig — (TIF) [file pone.0314457.s005.tif]

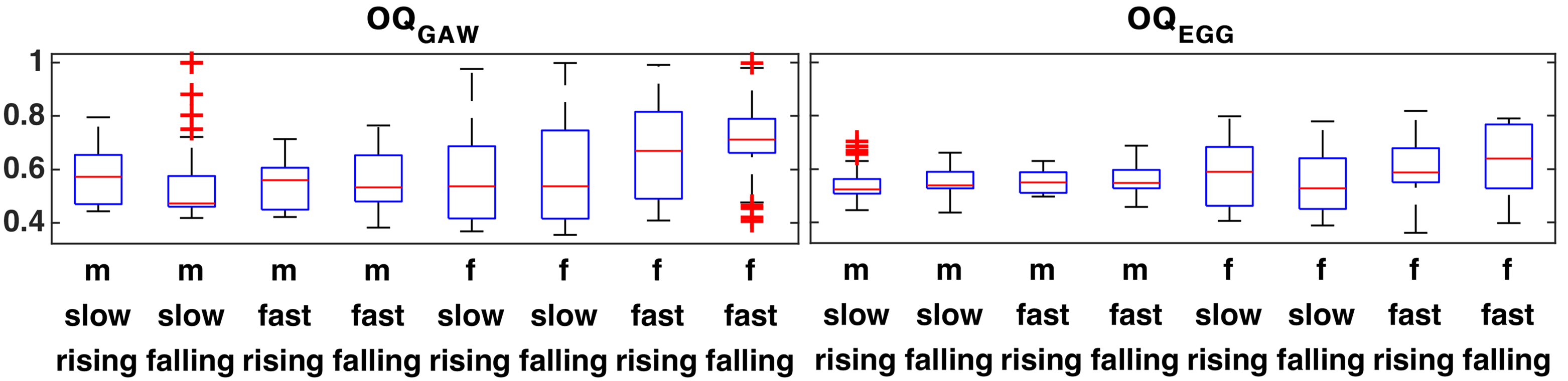

Supplement: S6 Fig — (TIF) [file pone.0314457.s006.tif]

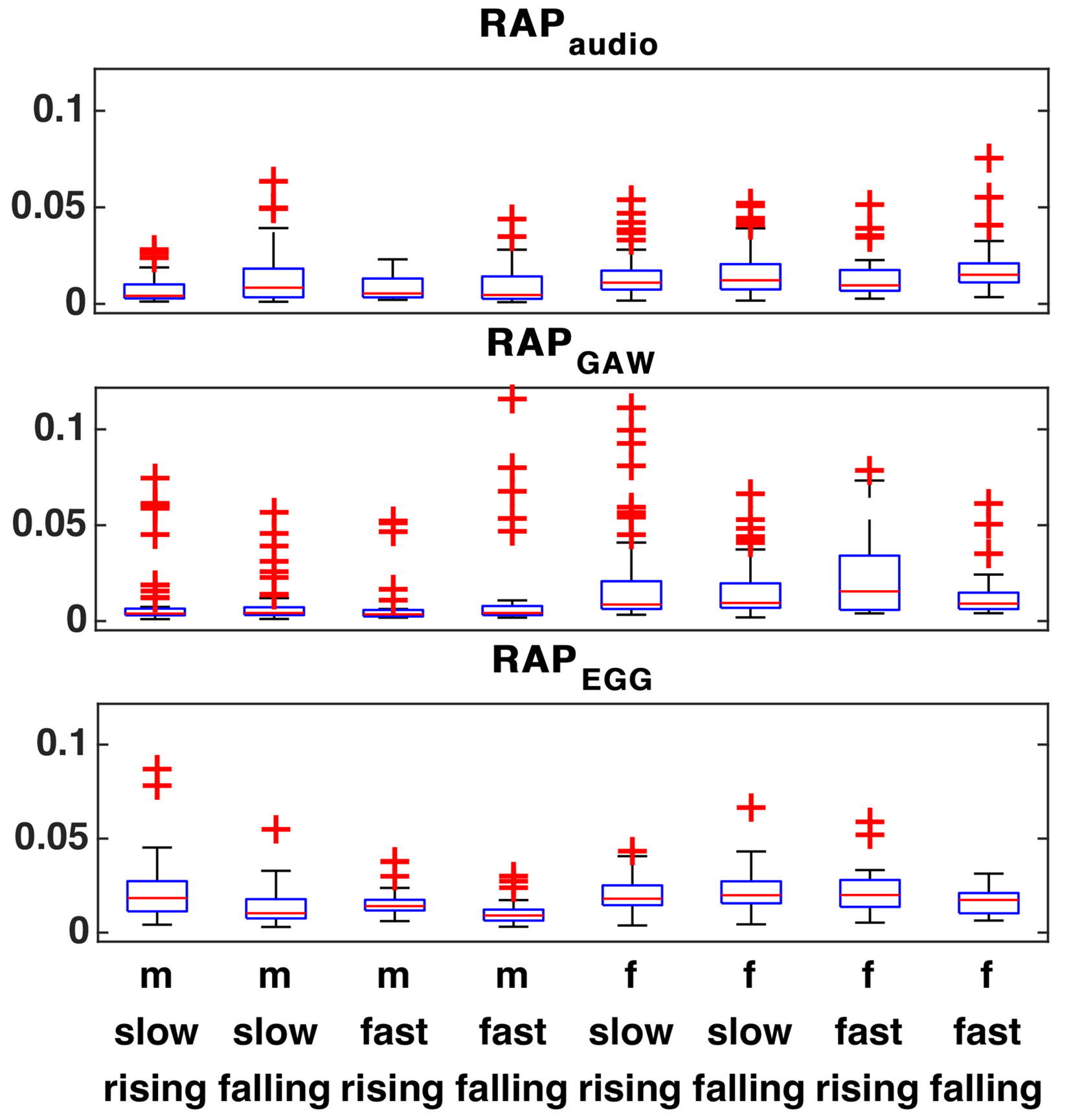

Supplement: S7 Fig — (TIF) [file pone.0314457.s007.tif]
