# Supplementary material for: Influence of Messa di Voce speed on vocal stability of untrained, healthy subjects
Source: PLoS One. 2025 Jan 30;20(1):e0314457. doi: 10.1371/journal.pone.0314457 (PMC11781711; doi:10.1371/journal.pone.0314457)
Supplement: S1 Text — Those formulas are also used in the Multi Signal Analyser [37]. (PDF) [file pone.0314457.s018.pdf]

FRIEDRICH-ALEXANDER UNIVERSITÄT ERLANGEN-NÜRNBERG

Universitätsklinikum Erlangen

Glottis Analysis Tools 2020

- User guide -

IMPORTANT NOTE: You cannot have two versions of GAT installed at the same time on one computer.

We have reworked core aspects of the code at the foundation of GAT for the new version. This means:

1. Segmentations created with the previous version can be used in the new version
2. Please **do not mix parameter computations** from previous versions with the new version

While we provide backward compatibility towards GAT 2018 segmentation files from earlier version can not longer be opened with GAT 2020. Furthermore we would strongly advise to avoid mixing the computations of parameters between GAT 2018 and GAT 2020 to ensure consistency. Hence, we recommend to wait with updating your current version until your studies are finished or to recompute the parameters with the new version.

Division for Phoniatrics and Pedaudiology at the ENT Department,
University Hospital Erlangen
Waldstr. 1
91054 Erlangen, Germany

3 Data Analysis

3.1 Fundamental frequency

These parameters can be calculated for the following input data:

- Glottal area waveform (GAW)
- Audio signal
- Glottal trajectories

The following parameters are calculated:

1. **Fundamental frequency**
   Fundamental-Freq $(\text{Hz})_i$ - oscillation frequency of the ith cycle in Hz (eq. 2).
2. **Cycle Duration**
   Cycle-duration $(ms)_i$ - duration of the ith cycle in ms.
3. **Maximum Harmonic**
   max-Harmonic (Hz) - maximum occurring harmonic frequency (a multiple of the fundamental frequency) in Hz.
4. **Minimum Subharmonic**
   min-Subharmonic (Hz) - minimum occurring subharmonic frequency (the fundamental frequency is a multiple of this frequency) in Hz.

3.1.1 Fundamental frequency: Determination

The fundamental frequency f_0^i of the ith cycle is calculated as follows:

$$f_0^i\ (\text{Hz}) = \frac{\text{Sampling rate (Hz)}}{T_i}, \tag{2}$$

where T_i is duration of the ith cycle in samples.

The cycles, and thereby the global fundamental frequency (f_0) will be detected using the following method:

> **Spectrum-based detection**: The Fourier spectrum of the given signal or signal window is calculated and the local maximum plus possible side peaks in the given *Tolerance range* (sec. 2.2.2 - C1) are detected. For every peak greater than the *Magnitude tolerance limit* (sec. 2.2.2 - C2) possible cycles are calculated. Subsequently the correlation between these cycles based on a cosine similarity like approach is calculated. The fundamental frequency which results in the cycles with the highest correlation is chosen as the final fundamental frequency f_0
>
> Note: the paramter Fundamental-Freq $(\text{Hz})_i$ is calculated based on the cycels which are determined by using the global f_0. Therefore the mean of all f_0^i and the directly calculated f_0 can differ marginally. Since the maximum and minimum harmonics are calculated by using directly f_0 they also tend to differ slightly from mean f_0^i if no significant harmonics or subharmonics in the signal exist.

3.2 Perturbation

The perturbation parameters quantify the average variability of the measured values.
These parameters can be calculated for the following signals:

- Glottal area waveform (GAW)
- Audio signal
- Glottal trajectories

Perturbation parameters will be calculated for the following signal features:

- Amplitude (sec. 3.2.1)
- Period: duration of the oscillation cycles (sec. 3.2.2)
- Energy (sec. 3.2.3)

each perturbation parameter is *cycle based*.

3.2.1 Amplitude

Further parameters:

- $A(i)$ - dynamic range $(\max - \min)$ of the ith cycle,
- N - the number of analyzed cycles (equivalent to the number of elements in A).

The following *cycle based* amplitude-related parameters are calculated:

1. **mean Shimmer** [1, 2]

$$\text{Mean-Shim } (dB) = \frac{20}{N-1} \sum_{i=0}^{N-2} \left| \log_{10} \left[\frac{A(i)}{A(i+1)} \right] \right| . \tag{3}$$

2. **Shimmer (%)** [3] (**new version since GAT 2019**)

$$\text{Shim } (\%) = \frac{\frac{1}{N-1} \sum_{i=1}^{N-1} |A(i) - A(i-1)|}{\frac{1}{N} \sum_{i=0}^{N-1} A(i)} \cdot 100. \tag{4}$$

3. **APQ (Amplitude Perturbation Quotient)** [4, 5, 6]

$$\text{APQ } (\%) = \frac{1}{N-k} \sum_{i=\frac{k-1}{2}}^{N-\frac{k-1}{2}-1} \left| 1 - \frac{k \cdot A(i)}{\sum_{j=-\frac{k-1}{2}}^{\frac{k-1}{2}} A(i+j)} \right| \cdot 100, \tag{5}$$

where k represents the number of cycles considered for computation of the quotients:

- $k = 3$: APQ-3 (%)
- $k = 5$: APQ-5 (%)
- $k = 11$: APQ-11 (%)

4. **APF (Amplitude Perturbation Factor)** [4, 5, 6]

$$\text{APF } (\%) = \frac{1}{N-1} \sum_{i=1}^{N-1} \left| \frac{A(i) - A(i-1)}{A(i)} \right| \cdot 100. \tag{6}$$

5. **AVI (Amplitude Variability Index)** [2]

$$\text{AVI} = \log_{10} \left(1000 \cdot \frac{\frac{1}{N} \sum_{i=1}^{N} \left[A(i) - \bar{A} \right]^2}{\bar{A}^2} \right), \tag{7}$$

where $\bar{A}$ represents the mean amplitude for over all analyzed cycles.

3.2.2 Period

Further parameters:

- $p(i)$ - duration of the ith cycle in ms,
- N - the number of analyzed cycles (equivalent to the number of elements in p).

The following *cycle based* period-related parameters are calculated:

1. **mean Jitter** [1]

$$\text{Mean-Jitter } (ms) = \frac{\sum_{i=1}^{N-1} |p(i) - p(i-1)|}{N-1}. \tag{8}$$

2. **Jitter (%)** [1]

$$\text{Jitter (\%)} = \frac{\frac{1}{N-1} \sum_{i=1}^{N-1} |p(i) - p(i-1)|}{\frac{1}{N} \sum_{i=0}^{N-1} p(i)} \cdot 100. \tag{9}$$

3. **Jitter Ratio** [2]

$$\text{Jitter Ratio} = \frac{\frac{1}{N-1} \sum_{i=1}^{N-1} |p(i) - p(i-1)|}{\frac{1}{N} \sum_{i=0}^{N-1} p(i)} \cdot 1000. \tag{10}$$

4. **Jitter Factor** [2]

$$\text{Jitter-Factor} = \frac{\frac{1}{N-1} \sum_{i=1}^{N-1} |f_i - f_{i-1}|}{\frac{1}{N} \sum_{i=0}^{N-1} f_i} \cdot 100, \tag{11}$$

where $f_i = \frac{1}{p(i)}$ is the frequency of the ith cycle in Hz.

5. **PPQ (Period Perturbation Quotient)** [4, 5, 6]

$$\text{PPQ (\%)} = \frac{1}{N-k} \sum_{i=\frac{k-1}{2}}^{N-\frac{k-1}{2}-1} \left| 1 - \frac{k \cdot p(i)}{\sum_{j=-\frac{k-1}{2}}^{\frac{k-1}{2}} p(i+j)} \right| \cdot 100, \tag{12}$$

where k represents the number of cycles considered for the computation of the quotients:

- $k = 3$: PPQ-3 (%)
- $k = 5$: PPQ-5 (%)
- $k = 11$: PPQ-11 (%)

6. **PPF (Period Perturbation Factor)** [4, 5, 6]

$$\text{PPF (\%)} = \frac{1}{N-1} \sum_{i=1}^{N-1} \left| \frac{p(i) - p(i-1)}{p(i)} \right| \cdot 100. \tag{13}$$

7. **RAP (Relative Average Perturbation)**

V1. [1]

$$\text{RAP-v1} = \frac{\sum_{i=1}^{N-2} \left| \frac{p(i-1)+p(i)+p(i+1)}{3} - p(i) \right|}{\sum_{i=0}^{N-1} p(i)}, \tag{14}$$

V2. [7, 2]

$$\text{RAP-v2} = \frac{\frac{1}{N-2} \sum_{i=1}^{N-2} \left| \frac{p(i-1)+p(i)+p(i+1)}{3} - p(i) \right|}{\frac{1}{N} \sum_{i=0}^{N-1} p(i)}. \tag{15}$$

8. **PVI (Period Variability Index)** [2]

$$\text{PVI} = 1000 \cdot \frac{\frac{1}{N} \sum_{i=1}^{N} [p(i) - \bar{p}]^2}{\bar{p}^2}, \tag{16}$$

where $\bar{p}$ represents the mean cycles duration.

3.2.3 Energy

Further parameters:

- $E(i)$ - signal energy within the ith cycle,
- N - the number of analyzed cycles (equivalent to the number of elements in E).

The following *cycle based* energy-related parameters are calculated:

1. **EPQ (Energy Perturbation Quotient)** [4, 5, 6]

$$\text{EPQ (\%)} = \frac{1}{N-k} \sum_{i=\frac{k-1}{2}}^{N-\frac{k-1}{2}-1} \left| 1 - \frac{k \cdot E(i)}{\sum_{j=-\frac{k-1}{2}}^{\frac{k-1}{2}} E(i+j)} \right| \cdot 100, \tag{17}$$

where k represents the number of cycles considered for computation of quotients:

- $k = 3$: EPQ 3 (%)
- $k = 5$: EPQ-5 (%)
- $k = 11$: EPQ-11 (%)

2. **EPF (Energy Perturbation Factor)** [4, 5, 6]

$$\text{EPF (\%)} = \frac{1}{N-1} \sum_{i=1}^{N-1} \left| \frac{E(i) - E(i-1)}{E(i)} \right| \cdot 100. \tag{18}$$

3.3 Noise

These measures can be calculated for the following signals:

- Glottal area waveform (GAW)
- Audio signal
- Glottal trajectories

Further parameters:

- $F(k)$ - k^{th} coefficient of the Fourier transform of the signal ($F(0)$ is the DC component),
- $C(k)$ - k^{th} Cepstrum coefficient [8]:

$$C(\omega) = 10 \cdot \log_{10}\left(\left|\mathcal{F}\{10 \cdot \log_{10}(|F(\omega)|^2)\}\right|^2\right), \tag{19}$$

- ω_0 - index of the Fourier coefficient that represents the fundamental frequency (f_0),
- H_{max} - maximum order of the harmonics of f_0,
- ω_{min} - index of the Fourier coefficient that represents the minimum occurring subharmonic for f_0.
- $f_{sampling}$ - sampling rate of the data.

The following parameters are calculated:

1. **Harmonics Intensity [litGAT:Hiraoka1984]**

   The parameter Harmonics Intensity is *window based*. The used window size can be influenced by the Fourier transformation settings (sec. 2.2.2 - C9 - C11)

$$\text{Harmonics-Intensity (\%)} = 100 \cdot \frac{\sum_{n=2}^{H_{max}} |F(n \cdot \omega_0)|}{\sum_{\omega \geq 1} |F(\omega)|}. \tag{20}$$

2. **Harmonics-to-Noise Ratio (HNR)** [9]

   The parameter HNR is *cycle based*.

$$\text{HNR } (dB) = 10 \cdot \log_{10}\left(\frac{H}{N}\right), \tag{21}$$

   with

$$H = n \int_0^T f_A^2(t) dt,$$

$$N = \sum_{i=1}^{n} \int_0^{T_i} |f_A(t) - f_i(t)|^2 \, dt,$$

   where

   - n - number of considered cycles,
   - T_i - duration of the ith cycle in ms,

- $f_i(t)$ - i^{th} cycle of the signal ($0 \leq t \leq T_i$),
- $T = \max\limits_{1 \leq i \leq n}(T_i)$,
- $f_A(t)$ - averaged cycle of the signal

$$f_A(t) = \frac{1}{n} \cdot \sum_{i=1}^{n} f_i(t), \qquad 0 \leq t \leq T. \tag{22}$$

3. **Normalized Noise Energy (NNE)** [10]

The parameter NNE is *window based*. A fix windowing algorithm is used:

- The signal is separated in 40 ms frames with 20 ms overlap.
- For each frame the median duration of all cycles with their starting position within this frame is calculated.
- From the original signal starting at the starting position of the frame a slice of data is cutted.
- This slice is as long as seven times the median duration of the cycles in the current frame.
- The slice is hamming windowed and afterwards filled with zeros to a length of 102,4 ms.
- The fourier transform of these zeropadded windows is calculated.

For each of these Fourier windows, let $s(\tau)$ be the periodic component, and $w(\tau)$ the noise component of the discretized signal $f(\tau)$, respectively, then

$$\Downarrow$$

$$f(\tau) = s(\tau) + w(\tau), \qquad \tau = 1, \ldots, M.$$

Discrete Fourier transform $\Downarrow$

$$F(\omega) = S(\omega) + W(\omega), \qquad \omega = 0, \ldots, N-1.$$

This leads to:

$$\text{NNE }(dB) = 10 \cdot \log_{10} \left(\frac{\sum\limits_{\omega=\omega_{min}}^{\omega_{max}} \left| \hat{W}(\omega) \right|^2}{\sum\limits_{\omega=\omega_{min}}^{\omega_{max}} |F(\omega)|^2} \right), \tag{23}$$

where

- $\omega_{max} = \min(\lceil \frac{\min(0.5 \cdot f_{sampling}, 5000)}{f_{sampling}} \cdot N \rceil - 1, \frac{N}{2} - 1)$,
- $\omega_{min} = \lceil \frac{50}{f_{sampling}} \cdot N \rceil - 1$,

with

$$\left|\hat{W}(\omega)\right|^2 = \begin{cases} |F(\omega)|^2\,, & \omega \in D_i \\ \frac{1}{2}\left(\sum_{r\in D_i}\frac{|F(r)|^2}{N_i} + \sum_{r\in D_{i+1}}\frac{|F(r)|^2}{N_{i+1}}\right), & \omega \in P_i \end{cases},$$

where

- $\left|\hat{W}(\omega)\right|^2 \approx |W(\omega)|^2$ (Fourier transform of the noise component $w(\tau)$),
- $D_i = \left\{r : (i-1)\cdot\omega_0 + \frac{2N}{M} \le r \le i\cdot\omega_0 - \frac{2N}{M}\right\}$,
- $P_i = \left\{r : i\cdot\omega_0 - \frac{2N}{M} \le r \le i\cdot\omega_0 + \frac{2N}{M}\right\}$,
- $N_i = |D_i|$ (Cardinality), $\quad 1 \le i \le H_{max}$.

The mean of all windows is the returned NNE parameter. If more than two successive D_i regions cant be calculated because of too close peaks, the respective window is excluded from analysis.

4. **Spectral Flatness** [5]

   The parameter Spectral Flatness is *window based*. The used window size can be influenced by the Fourier transformation settings (sec. 2.2.2 - C9 - C11)

$$\text{Spectral-Flatness } (SFM) = \frac{20}{N}\cdot\left(\sum_{i=1}^{N/2}\log_{10}|F(\omega)|^2\right) - 10\cdot\log_{10}\left(\frac{2}{N}\cdot\sum_{i=1}^{N/2}|F(\omega)|^2\right). \tag{24}$$

5. **Glottal-to-Noise Excitation Ratio (GNE)** [4, 11, 5]

   The parameter GNE is *window based*. The following algorithm is used:

   i) Changing the sampling rate to 50 kHz by sinosoidal interpolation
   ii) High pass filtering of the considered signal using:
      - Filter order: 10
      - cutoff frequency: 10 Hz
      - phase shift: zero
      - Method: Butterworth
      - Window: entire signal
   iii) Changing the sampling rate to 10 kHz
   iv) Discarding the first 10 ms of data (because of filter effects)
   v) Inverse filtering of the considered signal (calculation of the linear-prediction error signal) using:
      - Predictor order: 13
      - Method: auto correlation
      - Prediction window: Hann-window (length 30 ms, overlap 20 ms)
   vi) Discarding the first 13 samples of data (because of LP-filter effects)

vii) Subdividing the linear-prediction error signal in windows (length 500 ms, overlap 250 ms)

viii) Calculating the Fourier transformation of the windows.

ix) Calculating the Hilbert envelopes $\mathcal{H}(f_c)$ (the absolute value of the complex analytic signal) of frequency bands with fixed bandwidth (B_f) and different center frequencies (f_c) for each window. The center frequencies are divided in a group of left (f_{cl}) and right (f_{cr}) center frequencies:

- F_s - sampling rate of the signal $= 10000$
- $B_f = 3000Hz$
- f_c - Step $= 100Hz$
- f_{cl} - Range (Hz): $\frac{B_f}{2} \leq f_{cl} \leq 2000Hz$
- f_{cr} - Range (Hz): $3000Hz \leq f_{cr} \leq 5000Hz - \frac{B_f}{2}$

  Note: every slice of the fft window is multiplied with a hann window before back transformation.

x) Calculating $\rho_{k,j} = \max\left\{\text{CrossCorrelation}\left[\mathcal{H}(f_{cl}^k), \mathcal{H}(f_{cr}^j)\right]\right\}, \quad k, j \in \{1, 2, 3, 4, 5, 6\}$

xi) $GNE_i = \max_{k,j}(\rho_{k,j})$ Whereas i is the ith 500 ms window
Note: As other fixed window size Parameters GNE will not be calculated for too short signals. The minimum length for which one GNE window is calculated is 531.3 ms.

6. **Waveform Matching Coefficient (WMC)** [5]

The parameter WMC is *window based*. A fix windowing algorithm is used:

- The mean Cycle duration of all detected cycles is calculated.
- The signal is divided in subvectors of this length.
- These subvectors are used for the following calculation.

Note: This preprocessing of the signal is necessary since the calculation of cosine similarity requires data vectors of equal length.

$$\text{max-WMC} = \max_{1 \leq i \leq n-1} \frac{\langle f_i(t), f_{i-1}(t) \rangle}{\|f_i(t)\|_2 \cdot \|f_{i-1}(t)\|_2}, \tag{25}$$

where

- n - number of considered subvectors,
- D - duration of each subvector in frames (average of all cycle lengths),
- $f_k(t)$ - k^{th} subvector of the signal ($0 \leq t \leq D$)

7. **Mean Waveform Matching Coefficient (MWMC)** [5]

The parameter MWMC is *window based*. A fix windowing algorithm is used:

- The mean Cycle duration of all detected cycles is calculated.
- The signal is divided in subvectors of this length.
- These subvectors are used for the following calculation.

Note: This preprocessing of the signal is necessary since the calculation of cosine similarity requires data vectors of equal length.

$$\text{mean-WMC} = \underset{1 \leq i \leq n-1}{\text{mean}} \frac{\langle f_i(t), f_{i-1}(t) \rangle}{\|f_i(t)\|_2 \cdot \|f_{i-1}(t)\|_2}, \tag{26}$$

where

- n - number of considered subvector,
- D - duration of each subvector in frames (average of all cycle lengths),
- $f_k(t)$ - k^{th} subvector of the signal ($0 \leq t \leq D$).

8. **Signal-to-Noise Ratio (SNR)**

V1. [12, 13]

The parameter SNR-V1 is *window based*. The following algorithm is used:

i) Separating the signal in 160 ms gaussian windows with 20 ms signal shift (140 ms overlap).

ii) Zeropadding the signal windows to a length of 320 ms.

iii) Calculating the Fourier transformation of each zeropadded window i: $(F(\omega)_i)$.

iv) Further calculations are only done with the left half of this spectrum.

v) Upscaling $F(\omega)_i : \mathrm{Length}\left(\hat{F}_i\right) = 4 \cdot \mathrm{Length}\left(F_i\right)$

vi) Low pass filtering $\hat{F}_i$ using:
- Filter order: 4
- cutoff frequency: $0.25 \cdot f_{sampling}\, Hz$
- phase shift: zero
- Method: Butterworth

vii) Calculating the positions of f_0 (ω_0) and all possible harmonic positions (ω_i) without considering a threshold.

viii) Calculating an approximation for the noise in between the harmonics for each window i:
- define the "valleys" between the harmonics (D_j):

$$f_{step} = \frac{1}{0.16s \cdot 8}$$

$$D_{j+1} = \left\{ r : \omega_j + \frac{12Hz}{f_{step}} \leq r \leq \omega_{j+1} - \frac{12Hz}{f_{step}} \right\}$$

- special case region before the harmonics:

$$D_0 = \left\{ r : 0 + \frac{12Hz}{f_{step}} \leq r \leq \omega_0 - \frac{12Hz}{f_{step}} \right\}$$

- special case region after the last harmonic:

$$D_{jmax+1} = \left\{ r : \omega_{jmax} + \frac{12Hz}{f_{step}} \leq r \leq \omega_{jmax} + \omega_0 - \frac{12Hz}{f_{step}} \right\}$$

- Averaging $\hat{W}_j = \frac{1}{N_j} \left(\sum_{r \in D_j} F(r) \right)$

  where N_j is the number of samples in region D_j

ix) Calculating the half-value widths of the harmonics

x) All harmonics with half-value widths outside the range of 12 Hz to 24 Hz are excluded.

xi) All harmonics with peak values less than 12 dB above the mean noise of both surrounding "valleys" ($\hat{W}_j$ and $\hat{W}_{j+1}$) are excluded.

xii) Choosing the half-value (B_0) width of the greatest harmonic (usually f_0).

xiii) Approximating the pure harmonic spectrum ($\hat{H}_i$), using the harmonics positions,their peak value and the half-value width as follows:

- Each remaining harmonic is represented by a Gaussian shaped function
- Each Gaussian function has the same maximum and maximum position than their corresponding harmonic
- Each Gaussian function has the half-value with B_0.
- a vector of zeros as long as the first half of the Fourier window is generated
- To this vector the Gaussian functions are added

xiv) Calculating the harmonic short-time energy of window i: $E_h^i = \sum_{\omega=0}^{\text{Length}(\hat{H})} \left|\hat{H}(\omega)_i\right|^2$

xv) Calculating the total short-time energy of window i: $E_t^i = \sum_{\omega=0}^{\text{Length}(\hat{F})} \left|\hat{F}(\omega)_i\right|^2$

xvi) $SNR - v2_i(dB) = \min\left(10 \cdot \log_{10}\left(\frac{E_t^i}{E_t^i - E_h^i}\right), 36dB\right)$

Note: In the original source this algorithm is repeated with a narrower B_0, if $E_h^i > E_t^i$ until $E_h^i < E_t^i$. In such cases our implementation returns the value NaN for the corresponding window.

V2. [14]

The parameter SNR-V2 is *window based*. The following algorithm is used:

i) Short-term inverse filtering of the signal $f(t)$ (calculation of the linear-prediction error signal $s(t)$) using:
   - Predictor order: 14
   - Method: auto correlation
   - Prediction window: Hamming-window (length 20 ms, no overlap.)

ii) Discarding the first 14 samples of $s(t)$ (because of LP-filter effects)

iii) Long-term inverse filtering of $s(t)$ (calculation of the linear-prediction error signal $n(t)$) using:
   - Prediction order: 3,
   - Method: auto correlation
   - Window for minimizing prediction error: 2.5 ms,

iv) Optimizing long term filter over 1.25 - 17.5 ms range before current prediction start-sample $s(t_{windowstart})$.

v) Predicting the following 2.5 ms of signal beginning with $s(t_{windowstart})$ with the long term prediction coefficients.

vi) Discarding the first 17.5 ms of $n(t)$ (because no long term prediction was made here)

vii) Discarding the first 3 samples of $n(t)$ (because of LP-filter effects)

viii) $f(\hat{t}) = f(t + 3 + 14 + \lceil f_{sampling} * 0.0175 \rceil)$ ($\left|\hat{f}\right| = |f| - 3 - 14 - \lceil f_{sampling} * 0.0175 \rceil$ (cardinality)).

ix) SNR-v2 $(dB) = 20 \cdot \log_{10}\left[\frac{\sqrt{\sum_{t=1}^{N}\left|\hat{f}(t)\right|^2}}{\sqrt{\sum_{t=1}^{N}|n(t)|^2}} - 1\right]$.

9. **Cepstral Peak Prominence (CPP)**

I. [15, 8]

The parameter CPP-I is *window based*. The used window size can be influenced by the Fourier transformation settings (sec. 2.2.2 - C9 - C11). The following algorithm is used:

i) Calculating the Cepstrum for the given Fourier window (eq. 19).

ii) Detecting the first Rahmonic.

iii) Calculating the regression line ($R(k)$) in the Range of 1 ms to half the length of the Cepstrum.

iv) Subtracting the maximum of the first Rahmonic from the corresponding value on the regression line: $CPP_i = C(r_0)^i - R(r_0)^i$

II. [15, 8]

The parameter CPP-II is *window based*. The used window size can be influenced by the Fourier transformation settings (sec. 2.2.2 - C9 - C11). The following algorithm is used:

i) Scaling the Fourier windows to Db

ii) Averaging all given rescaled Fourier windows to an average window ($\bar{F}_{log}$) if the have the same length.

iii) Calculating the Cepstrum: $C(\omega) = 10 \cdot \log_{10} \left(\left| \mathcal{F}\{\bar{F}(\omega)_{log}\} \right|^2 \right)$.

iv) Detecting the first Rahmonic.

v) Calculating the regression line ($R(k)$) in the Range of 1 ms to half the length of the Cepstrum.

vi) Subtracting the maximum of the first Rahmonic from the corresponding value on the regression line: $CPP = C(r_0) - R(r_0)$

3.4 Mechanical

These parameters can be calculated for the following signals:

- Glottal area waveform (GAW)
- Glottal trajectories

Further parameters:

- T_i - duration of the ith cycle in ms,
- A_i - dynamic range $(\max - \min)$ of the ith cycle,
- L_i - mean glottis length (distance between Anterior- and Posterior- points) for the ith cycle,
- $s(t)$ - absolute magnitude of the 1st derivative of the considered signal for i^{th} cycle ($t \subset T_i$).

The following *cycle based* mechanical parameters are calculated:

1. **Stiffness** [16]

$$\text{Stiffness} = \frac{\max_{t \subset T_i}(s(t))}{A_i}. \tag{27}$$

2. **Peak Closing Velocity** [17]

$$\text{Peak-Clossing-Velocity} = 2\pi \cdot \frac{A_i}{2 \cdot T_i}. \tag{28}$$

3. **Peak Acceleration** [17]

$$\text{Peak-Acceleration} = 4\pi^2 \cdot \frac{A_i}{2 \cdot T_i^2}. \tag{29}$$

4. **Amplitude-to-Length Ratio**

$$\text{Amplitude-Length-Ratio} = \frac{A_i}{L_i}. \quad (30)$$

3.5 Glottal area waveform

These parameters can be calculated for the following signals:

- Glottal area waveform (GAW)
- Glottal trajectories

Further parameters:

- T_i - duration of ith cycle in ms,
- t^i_{closed} - *closed phase duration*, defined when the glottis is closed (glottal area ≤ 0),
- t^i_{open} - *open phase duration*, defined when the glottis is open (glottal area > 0),
- $[C \rightarrow O]_i$ - opening part of open phase (closed-to-open) for i^{th} cycle,
- $[O \rightarrow C]_i$ - closing part of open phase (open-to-closed) for i^{th} cycle,
- GA_i - glottal area for i^{th} cycle
- A_i - Glottal area dynamic range $[\max(GA_i) - \min(GA_i)]$ for i^{th} cycle,

The Phases are dependent of the zero line, which is 0 by default. However, it can be adjusted using the settings (sec. 2.2.3 - C11).
Note: To avoid discretization gaps between the different cycles (i.e. if one cycle ends at datapoint x and the next one starts at datapoint x+1 the between this datapoints would be undefined), the phases for each cycle are calculated from its first datapoint up to the last plus one datapoint. The following *cycle based* quotients are calculated (fig. 9):

1. **Open Quotient** [2]

$$\text{Open-Quotient } (O_q) = \frac{t^i_{open}}{T_i}. \quad (31)$$

2. **Closing Quotient** [18]

$$\text{Closing-Quotient } (Cl_q) = \frac{[O \rightarrow C]_i}{T_i}. \quad (32)$$

3. **Speed Quotient** [2]

$$\text{Speed-Quotient } (S_q) = \frac{[C \rightarrow O]_i}{[O \rightarrow C]_i} \quad (33)$$

4. **Speed Index** [2]

$$\text{Speed-Index } (SI) = \frac{[C \rightarrow O]_i - [O \rightarrow C]_i}{t^i_{open}} = \frac{[C \rightarrow O]_i - [O \rightarrow C]_i}{[C \rightarrow O]_i + [O \rightarrow C]_i} = \frac{(S_q - 1)}{(S_q + 1)}. \quad (34)$$

5. **Rate Quotient** [2]

$$\text{Rate-Quotient } (R_q) = \frac{(t^i_{closed} + [C \rightarrow O]_i)}{[O \rightarrow C]_i}. \quad (35)$$

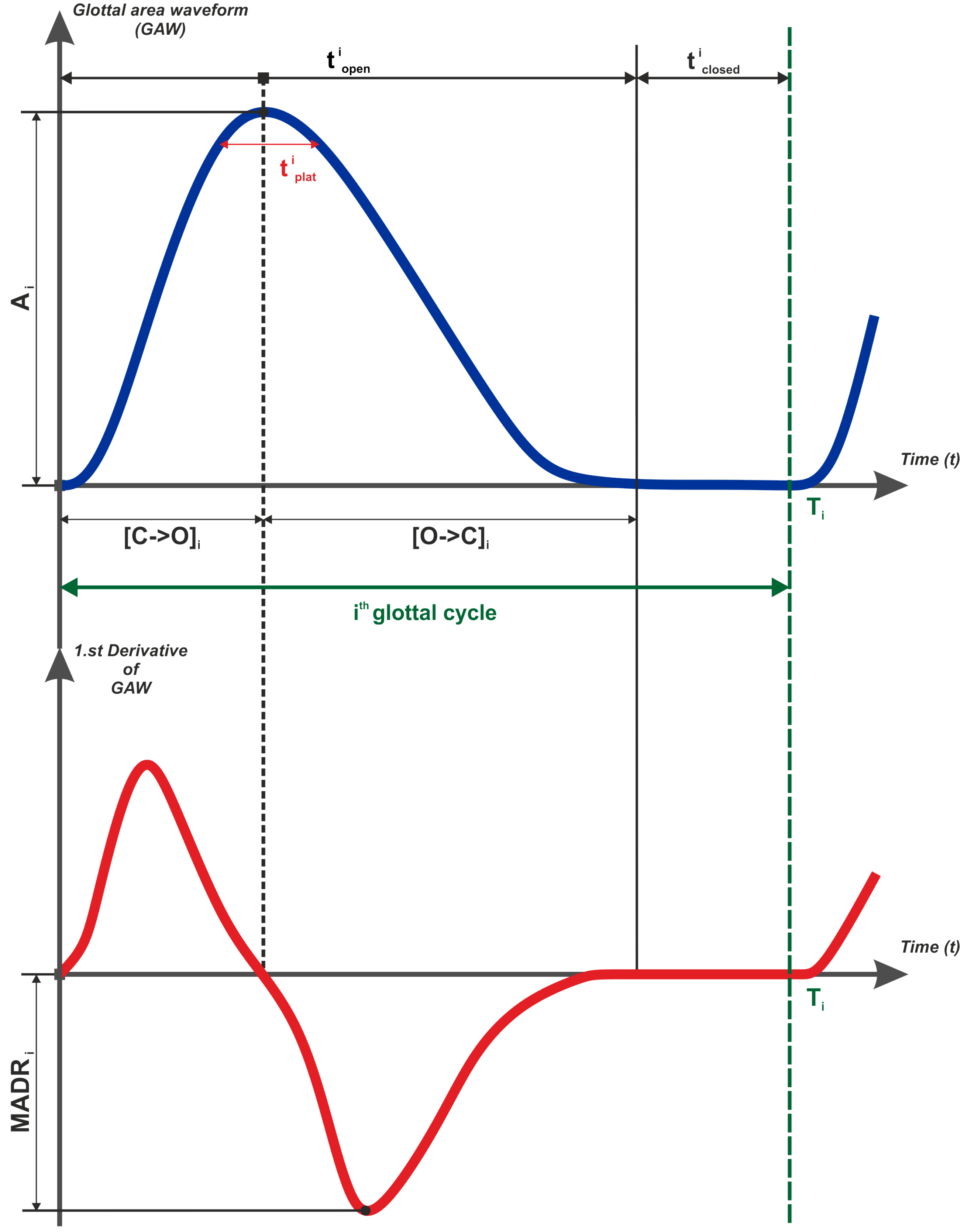

Figure 9 – *Visualization of Open Quotient, Closing Quotient, Speed Quotient, Speed Index, Rate Quotient, Asymmetry Quotient, Plateau Quotient, and GAW Derivatives parameters (sec. 3.5.2).*

6. **Asymmetry Quotient** [19]

$$\text{Asymmetry-Quotient } (A_q) = \frac{S_q}{1 + S_q}. \tag{36}$$

7. **Glottis Gap Index** [20]

$$\text{Glottis-Gap-Index } (GGI) = \frac{\min(GA_i)}{\max(GA_i)}, \tag{37}$$

8. **Plateau Quotient** [21]

$$\text{Plateau-Quotient } (P_q) = \frac{t^i_{plat}}{t^i_{open}}, \tag{38}$$

   where t^i_{plat} is the plateau phase duration of the ith cycle,
   i.e. the phase when the glottal area is greater than 95% of its maximum.
   this threshold value of 95% can be changed using (sec. 2.2.3 - C10)

9. **Glottal Area Index** [22]

$$\text{Glottal-Area-Index } (AC/OQ) = \frac{A_i}{\max(GA_i) \cdot O_q}, \tag{39}$$

3.5.1 Glottal area waveform: Periodicity

These parameters originally proposed by Qiu et al. [23] for the digital kymography were adapted to the signals generated with high speed imaging (fig. 10). Both parameters are *cycle based*.

1. **Amplitude periodicity** [23]

$$\text{Amplitude-Periodicity} = \frac{\min(A_i, A_{i+1})}{\max(A_i, A_{i+1})}. \tag{40}$$

2. **Time periodicity** [23]

$$\text{Time-Periodicity} = \frac{\min(T_i, T_{i+1})}{\max(T_i, T_{i+1})}. \tag{41}$$

3.5.2 Glottal area waveform: Derivatives

These parameters originally used for the glottal air flow [18, 24] were adapted to the signals generated from high speed imaging (fig. 9). Both parameters are *cycle based*.

1. **Maximum Area Declination Rate** [18, 24, 25, 26]

   Maximum-Area-Declination-Rate $(MADR)$ is defined as the absolute maximum amplitude of the negative peak of the 1st derivative of the considered signal (here: glottal area waveform or glottal trajectories).

2. **Amplitude Quotient** [24, 26]

$$\text{Amplitude-Quotient } (AQ) = \frac{A_i}{MADR_i}. \tag{42}$$

3.6 Symmetry

These parameters can be calculated for the following signals:

- Glottal area waveform (GAW)

- Glottal trajectories

Symmetry parameters are separated into following the subsets:

- Unilateral symmetry parameters (sec. 3.6.1)
- Lateral symmetry parameters: not suitable for between-group comparison (sec. 3.6.2)

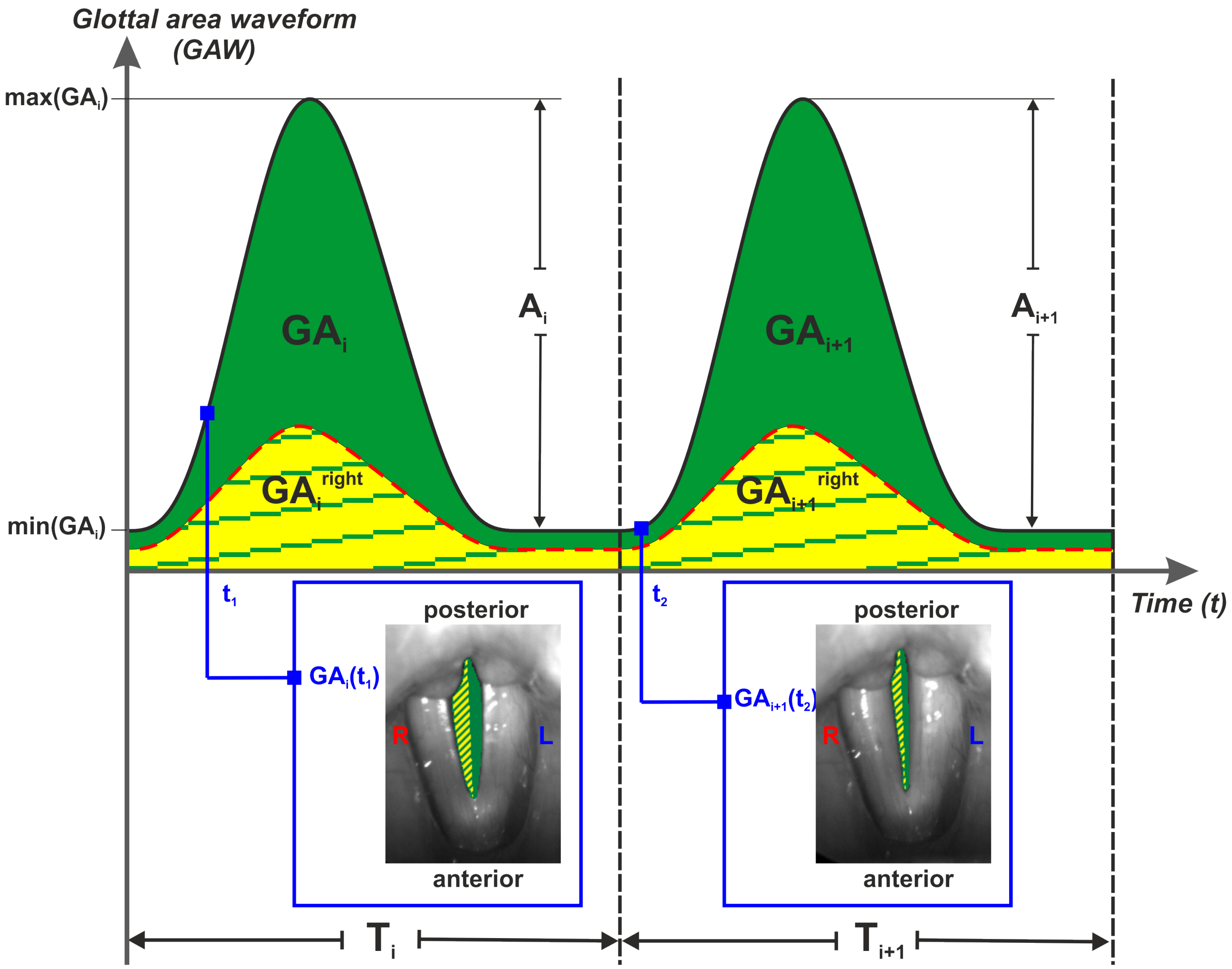

Figure 10 *– Visualization of the Glottis Gap Index, GAW Periodicity parameters (sec. 3.5.1) and Symmetry parameters (sec. 3.6).*

Furthermore:

- T_i - duration of the ith cycle in ms,
- GA_i - glottal area waveform of the ith cycle,
- A_i - glottal area dynamic range $[\max(GA_i) - \min(GA_i)]$ of the ith cycle,
- L - left side, R - right side.

3.6.1 Symmetry: Unilateral

The following unilateral *cycle based* symmetry parameters are calculated (fig. 10):

1. **Phase Asymmetry Index** [23, 20]

$$\text{Phase-Asymmetry-Index} = \frac{\left|t_i^L(\max) - t_i^R(\max)\right|}{T_i}, \tag{43}$$

where $t_i^{side}(\max)$ is the time at which the glottal area of the left/right side in the ith cycle is maximal.

2. **Spatial Symmetry Index** (for GAW only!) [20]

$$\text{Spatial-Symmetry-Index} = \frac{\left| \sum_{t \subset T_i} GA_i^L(t) - \sum_{t \subset T_i} GA_i^R(t) \right|}{\sum_{t \subset T_i} GA_i(t)}. \tag{44}$$

3. **Dynamic Range Symmetry Index**

$$\text{DynamicRange-Symmetry-Index} = \frac{\min(A_i^L, A_i^R)}{\max(A_i^L, A_i^R)}. \tag{45}$$

4. **Amplitude Symmetry Index**

$$\text{Amplitude-Symmetry-Index} = \frac{\min\left(\max[GA_i^L], \max[GA_i^R]\right)}{\max\left(\max[GA_i^L], \max[GA_i^R]\right)}. \tag{46}$$

5. **Waveform Symmetry Index** [27]

$$\text{Waveform-Symmetry-Index} = 0.5 \cdot \left[1 + \frac{\langle GA_i^L, GA_i^R \rangle}{\|GA_i^L\|_2 \cdot \|GA_i^R\|_2}\right]. \tag{47}$$

3.6.2 Symmetry: Lateral

In the *Glottis Analysis Tools* the following lateral *cycle based* symmetry parameters are calculated (fig. 10):

1. **Phase Asymmetry** [23]

$$\text{Phase-Asymmetry*} = \frac{t_i^L(\max) - t_i^R(\max)}{T_i}, \tag{48}$$

where $t_i^{side}(\max)$ is the time at which the glottal area of the left/right side in the ith cycle is maximal.

2. **Spatial Symmetry** (for GAW only!)

$$\text{Spatial-Symmetry*} = \frac{\sum_{t \subset T_i} GA_i^L(t) - \sum_{t \subset T_i} GA_i^R(t)}{\sum_{t \subset T_i} GA_i(t)}. \tag{49}$$

3. **Dynamic Range Symmetry**

$$\text{DynamicRange-Symmetry*} = \frac{A_i^L}{A_i^R}. \tag{50}$$

4. **Amplitude Symmetry**

$$\text{Amplitude-Symmetry*} = \frac{\max(GA_i^L)}{\max(GA_i^R)}. \tag{51}$$

3.7 Phonovibrogram

These parameters can only be calculated for the Phonovibrogram (PVG).
The following*cycle based* PVG parameters are calculated:

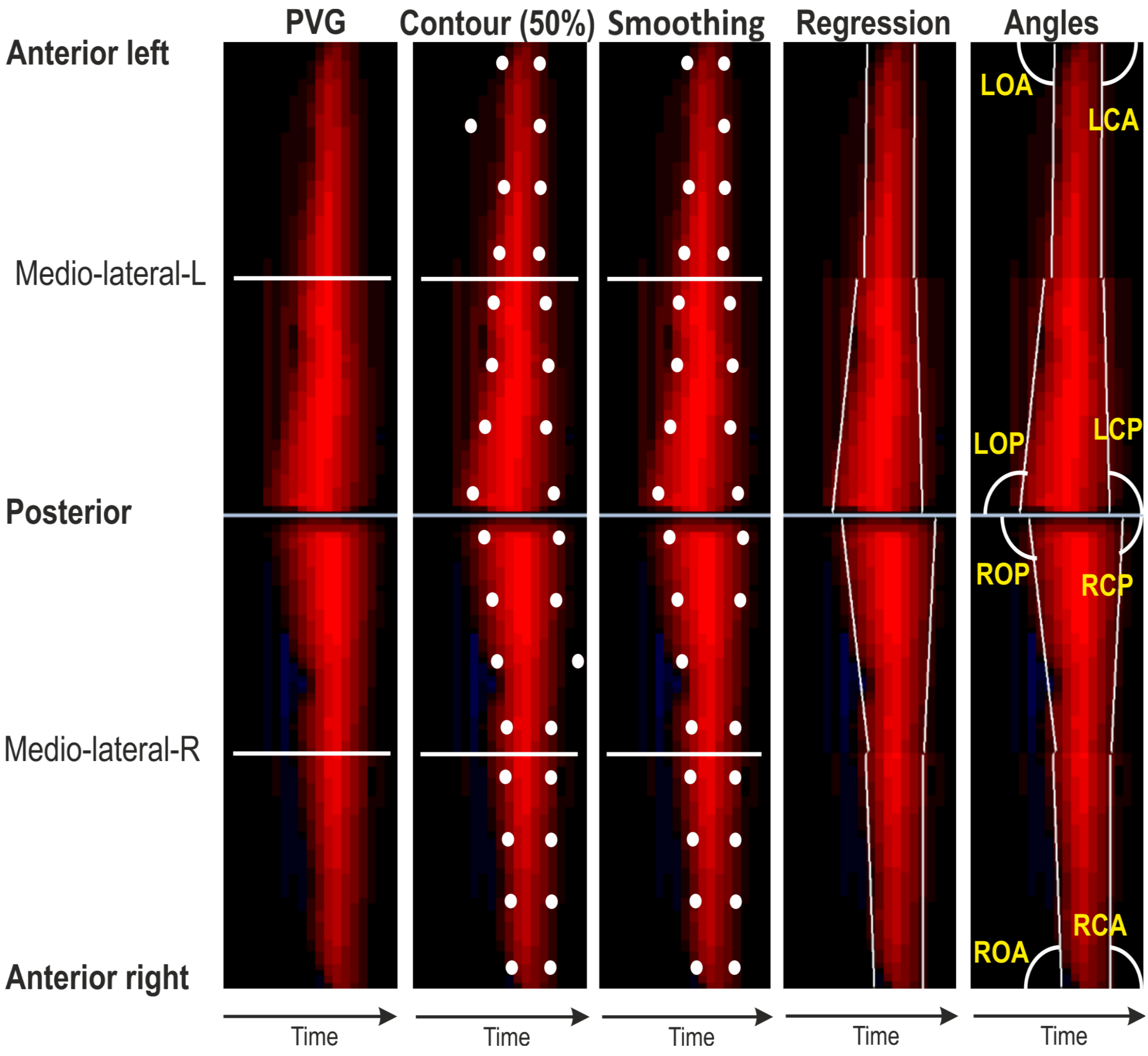

***Figure 11** – Visualization of the PVG Contour Angles.*

1. **Contour Angles** (fig. 11) [28, 29, 30]
   Contour-Angles (deg) are calculated in both anterior and posterior parts during opening as well as closing of the vocal folds for the left and right side of the PVG, respectively. Figure 11 roughly illustrates the calculation algorithm of the contour angles.
   Therein, $CA_i^{side,Item}$ denotes the Contour-Angles of the ith cycle,
   where $side$ represents the corresponding side of the PVG:

   - L - Left side,
   - R - Right side,

   and $Item$ indicates the position of the related Contour-Angle:

   - $Item = OA$: Opening - Anterior,

- $Item = OP$: Opening - Posterior,
- $Item = CA$: Closing - Anterior,
- $Item = CP$: Closing - Posterior.

2. **Contour Angles Symmetry Index** [31]

$$\text{ContourAngles-Symmetry-Index}_{Item} = \frac{\min(CA_i^{L,Item}, CA_i^{R,Item})}{\max(CA_i^{L,Item}, CA_i^{R,Item})}. \quad (52)$$

3. **Contour Angles Symmetry** (Not suitable for between-group comparison!) [31]

$$\text{ContourAngles-Symmetry*}_{Item} = \frac{CA_i^{L,Item}}{CA_i^{R,Item}}. \quad (53)$$

8 Literature

[1] S. Bielamowicz, J. Kreiman, B. R. Gerratt, M. S. Dauer, and G. S. Berke. “Comparison of voice analysis systems for perturbation measurement.” eng. In: *J Speech Hear Res* 39.1 (1996), pp. 126–134.

[2] R. J. Baken and R. F. Orlikoff. *Clinical measurement of speech and voice*. Second edition. Cengage Learning, 1999.

[3] I. R. Titze, Y. Horii, and R. C. Scherer. “Some technical considerations in voice perturbation measurements”. eng. In: *Journal of Speech, Language and Hearing Research* 30.2 (1987), pp. 252–260.

[4] D. Michaelis. “Das Göttinger Heiserkeits-Diagramm-Entwicklung und Prüfung eines akustischen Verfahrens zur objektiven Simmgütebeurteilung pathologischer Stimmen”. PhD thesis. Georg-August-Universität Göttingen, Mathematisch-Naturwissenschaftlich Fakultät, 1999.

[5] J. Lessing. “Entwicklung einer Klassifikationsmethode zur akustischen Analyse fortlaufender Sprache unterschiedlicher Stimmgüte mittels Neuronaler Netze und deren Anwendung”. PhD thesis. Georg-August-Universität Göttingen, Mathematisch-Naturwissenschaftlich Fakultät, 2007.

[6] H. Kasuya, Y. Endo, and S. Salin. “Novel Acoustic Measurements of Jitter and Shimmer Characteristics from Pathological Voice”. In: *EUROSPEECH’93* 1 (1993), pp. 1973–1976.

[7] Y. Koike. “Application of Some Acoustic Measures for the Evaluation of Laryngeal Dysfunction”. In: *Studia Phonoligica* 7 (1973), pp. 17–23.

[8] J. Hillenbrand and R. A. Houde. “Acoustic Correlates of Breathy Vocal Quality: Dysphonic Voices and Continuous Speech”. In: *Journal of Speech and Hearing Research* 39 (1996), pp. 311–321.

[9] E. Yumoto, W. J. Gould, and T. Baer. “Harmonics-to-noise ratio as an index of the degree of hoarseness.” eng. In: *J Acoust Soc Am* 71.6 (1982), pp. 1544–1549.

[10] H. Kasuya, S. Ogawa, K. Mashima, and S. Ebihara. “Normalized noise energy as an acoustic measure to evaluate pathologic voice.” eng. In: *J Acoust Soc Am* 80.5 (1986), pp. 1329–1334.

[11] D. Michaelis, M. Fröhlich, and H. W. Strube. “Selection and combination of acoustic features for the description of pathologic voices.” eng. In: *J Acoust Soc Am* 103.3 (1998), pp. 1628–1639.

[12] F. Klingholz. “The Measurement of the signal-to-noise ratio (SNR) in Continuos Speech”. In: *Speech Communication* 6 (1987), pp. 15–26.

[13] F. Klingholz. “Acoustic representation of speaking-voice quality.” In: *J Voice* 4.3 (1990), pp. 213–219.

[14] Y. Qi, R. E. Hillman, and C. Milstein. “The estimation of signal-to-noise ratio in continuous speech for disordered voices.” eng. In: *J Acoust Soc Am* 105.4 (1999), pp. 2532–2535.

[15] J. Hillenbrand, R. A. Cleveland, and R. L. Erickson. “Acoustic correlates of breathy vocal quality.” eng. In: *J Speech Hear Res* 37.4 (1994), pp. 769–778.

[16] K. G. Munhall, D. J. Ostry, and A. Parush. “Characteristics of velocity profiles of speech movements.” eng. In: *J Exp Psychol Hum Percept Perform* 11.4 (1985), pp. 457–474.

[17] I. R. Titze. “Mechanical stress in phonation.” eng. In: *J Voice* 8.2 (1994), pp. 99–105.

[18] E. B. Holmberg, R. E. Hillman, and J. S. Perkell. “Glottal airflow and transglottal air pressure measurements for male and female speakers in soft, normal, and loud voice.” eng. In: *J Acoust Soc Am* 84.2 (1988), pp. 511–529.

[19] N. Henrich, G. Sundin, D. Ambroise, C. d’Alessandro, M. Castellengo, and B. Doval. “Just noticeable differences of open quotient and asymmetry coefficient in singing voice.” eng. In: *J Voice* 17.4 (2003), pp. 481–494.

[20] R. Patel, D. Dubrovskiy, and M. Döllinger. “Characterizing vibratory kinematics in children and adults with high-speed digital imaging.” eng. In: *J Speech Lang Hear Res* 57.2 (2014), S674–S686.

[21] D. D. Mehta, M. Zaéartu, T. F. Quatieri, D. D. Deliyski, and R. E. Hillman. “Investigating acoustic correlates of human vocal fold vibratory phase asymmetry through modeling and laryngeal high-speed videoendoscopy.” eng. In: *J Acoust Soc Am* 130.6 (2011), pp. 3999–4009.

[22] G. Chen, J. Kreiman, B. R. Gerratt, J. Neubauer, Y.-L. Shue, and A. Alwan. “Development of a glottal area index that integrates glottal gap size and open quotient.” eng. In: *J Acoust Soc Am* 133.3 (2013), pp. 1656–1666.

[23] Q. Qiu, H. K. Schutte, L. Gu, and Q. Yu. “An automatic method to quantify the vibration properties of human vocal folds via videokymography.” eng. In: *Folia Phoniatr Logop* 55.3 (2003), pp. 128–136.

[24] P. Alku, M. Airas, E. Björkner, and J. Sundberg. “An amplitude quotient based method to analyze changes in the shape of the glottal pulse in the regulation of vocal intensity.” eng. In: *J Acoust Soc Am* 120.2 (2006), pp. 1052–1062.

[25] I. R. Titze. “Theoretical analysis of maximum flow declination rate versus maximum area declination rate in phonation.” eng. In: *J Speech Lang Hear Res* 49.2 (2006), pp. 439–447.

[26] R. R. Patel, D. Dubrovskiy, and M. Döllinger. "Measurement of glottal cycle characteristics between children and adults: physiological variations." eng. In: *J Voice* 28.4 (2014), pp. 476–486.

[27] P.-N. Tan, M. Steinbach, and V. Kumar. *Introduction to Data Mining*. Addison-Wesley, 2005.

[28] D. Voigt, M. Döllinger, A. Yang, U. Eysholdt, and J. Lohscheller. "Automatic diagnosis of vocal fold paresis by employing phonovibrogram features and machine learning methods." eng. In: *Comput Methods Programs Biomed* 99.3 (2010), pp. 275–288.

[29] D. Voigt, M. Döllinger, T. Braunschweig, A. Yang, U. Eysholdt, and J. Lohscheller. "Classification of functional voice disorders based on phonovibrograms." eng. In: *Artif Intell Med* 49.1 (2010), pp. 51–59.

[30] M. Döllinger, J. Lohscheller, A. McWhorter, and M. Kunduk. "Variability of normal vocal fold dynamics for different vocal loading in one healthy subject investigated by phonovibrograms." eng. In: *J Voice* 23.2 (2009), pp. 175–181.

[31] M. Döllinger, D. Dubrovskiy, and R. Patel. "Spatiotemporal analysis of vocal fold vibrations between children and adults." eng. In: *Laryngoscope* 122.11 (2012), pp. 2511–2518.
